# Supplementary material for: Survival probabilities of thornback skate (Raja clavata) and spotted skate (Raja montagui) discarded by tickler chain beam trawl, pulse trawl, and flyshoot fisheries
Source: PLoS One. 2024 Dec 19;19(12):e0314032. doi: 10.1371/journal.pone.0314032 (PMC11658617; doi:10.1371/journal.pone.0314032)
Supplement: S1 Table — (DOCX) [file pone.0314032.s001.docx]

**S1 Table. Gear specifics of the pulse trawlers used for the discards survival trips.**

| Specifics | | Vessel 1 | Vessel 2 | Vessel 3 |
| --- | --- | --- | --- | --- |
| Beam (wing) | Width (m) | 12 | 12 | 12 |
|  | Length (m) | 1.1 | 1.1 | 1.1 |
|  | Total weight (kg) | 2600 | 2740 | 2300 |
| False ground rope | Type | Rubber discs | Rubber discs | Rubber discs |
|  | Length (m) | 11.7 | 11 | 11.8 |
|  | Diameter (mm) | 220 | 120 | 120 |
|  | Total weight (kg) | 110 | 140 | 80 |
| Electrodes | Number | 22 | 24 | 26 |
|  | Type | HFK | HFK | HFK |
|  | Total length (m) | 7.5 | 7.2 | 7.4 |
|  | Distance between electrodes (cm) | 40.0 | 42.5 | 45.0 |
|  | Length electrodes on seabed (pulse field) (m) | 3.0 | 3.2 | 4.4 |
| Conductor elements | Number | 11 | 10 | 12 |
|  | Diameter (mm) | 35 | 28 | 33 |
|  | Length (mm) | 130 | 130 | 134 |
|  | Distance between elements (mm) | 220 | 210 | 200 |
| Pulse | Power (kW/m) | 6.0 | 5.3 | 7.3 |
|  | Width (µs) | 340 | 390 | 330 |
|  | Frequency (Hz) | 60 | 45 | 60 |
|  | Peak voltage over electrode (V) | 60 | 60 | 60 |
|  | Maximum exposure to pulse field (s) | 1.2 | 1.3 | 1.7 |
| Trawl | Total length (m) | 34 | 30 | 34 |
|  | Mesh size cod-end (mm) | 80 | 80 | 80 |
|  | Twine cod-end | Double knotted | Double knotted | Double knotted |
|  | Twine thickness (mm) | 4 | 3 | 3 |
