# Supplementary material for: Survival probabilities of thornback skate (Raja clavata) and spotted skate (Raja montagui) discarded by tickler chain beam trawl, pulse trawl, and flyshoot fisheries
Source: PLoS One. 2024 Dec 19;19(12):e0314032. doi: 10.1371/journal.pone.0314032 (PMC11658617; doi:10.1371/journal.pone.0314032)
Supplement: S2 Table — (DOCX) [file pone.0314032.s002.docx]

S2 Table. Gear specifics of the beam trawler used for the survival trips.

| Beam | Width (m) | 1 |
| --- | --- | --- |
|  | Length (m) | 12 |
| Ground rope | Length (m) | 37 |
|  | Diameter chain (mm) | 24 |
|  | Length central rubber section ground rope (m) | 7 |
| Chains | Tickler chains (n/gear) | 8 |
|  | Net ticklers (n/gear) | 14 |
|  | Total weight (ton) | 2x 2.3 |
| Trawl | Total length (m) | 40 |
|  | Mesh size cod-end (mm) | 80 |
|  | Height (m) | 0.6 |
