# Supplementary material for: Survival probabilities of thornback skate (Raja clavata) and spotted skate (Raja montagui) discarded by tickler chain beam trawl, pulse trawl, and flyshoot fisheries
Source: PLoS One. 2024 Dec 19;19(12):e0314032. doi: 10.1371/journal.pone.0314032 (PMC11658617; doi:10.1371/journal.pone.0314032)
Supplement: S3 Table — (DOCX) [file pone.0314032.s003.docx]

**S3 Table. Gear specifics of flyshooter used for the survival trips.**

| Trawl | Height (m) | 10 |
| --- | --- | --- |
|  | Cod end mesh size (mm) | 80 |
| Flyshoot rope | Length (m) | 2x 2900 |
|  | Diameter (mm) | 50 |
| Ground rope | Weight (kg/m) | 1.85 |
|  | Length (m) incl. chain & sweeps | 146.7 |
|  | Disc diameter (mm) | 280 |
| Escape panel | North sea | Yes |
|  | Eastern Channel | No |
|  | Mesh size (mm) | 110 |
