# Supplementary material for: Survival probabilities of thornback skate (Raja clavata) and spotted skate (Raja montagui) discarded by tickler chain beam trawl, pulse trawl, and flyshoot fisheries
Source: PLoS One. 2024 Dec 19;19(12):e0314032. doi: 10.1371/journal.pone.0314032 (PMC11658617; doi:10.1371/journal.pone.0314032)
Supplement: Attachment — Submitted filename: PONE-D-24-20479_reviewer_REV1.pdf [file pone.0314032.s009.pdf]

PLOS ONE

Survival probabilities of thornback and spotted rays discarded by tickler chain beam trawl, pulse beam trawl and flyshoot fisheries

--Manuscript Draft--

**Manuscript Number:**	PONE-D-24-20479
**Article Type:**	Research Article
**Full Title:**	Survival probabilities of thornback and spotted rays discarded by tickler chain beam trawl, pulse beam trawl and flyshoot fisheries
**Short Title:**	Discards survival of rays
**Corresponding Author:**	Edward Schram, Ph.D.<br>Wageningen Marine Research<br>Ijmuiden, NETHERLANDS
**Keywords:**	Discards survival, rays, North Sea, landing obligation, fisheries
**Abstract:**	To measure discards survival probabilities of thornback (Raja clavata) and spotted ray (Raja montagui) we conducted five trips with a tickler chain beam trawler during which 183 thornback and 136 spotted rays were sampled, nine trips with pulse beam trawlers during which 94 thornback rays were sampled and four trips with a flyshooter during which 137 thornback and 24 spotted rays were sampled. Survival probabilities were measured by captive observation for 15 to 25 days post catch. All fishery operations were conducted in the southern North Sea (ICES division 27.4.c) and in the Eastern English Channel (ICES division 27.7.d) according to the regular commercial practices of the fishing vessels. Trips were spread out over the seasons to account for the effect of variable environmental and fishing conditions on discards survival. Operational and environmental conditions during sea trips were recorded. For beam trawling survival probabilities (95% CI) were 50% (43-57) and 44% (37-54) for thornback and spotted ray. For pulse trawling survival probability of thornback was 54% (40-65). For flyshooting survival probabilities were higher: 80% (73-87) for thornback and 75% (60-94) for spotted ray. Survival probabilities were significantly affected by gear, catch processing time, water temperature, wave height and the interaction between water temperature and wave height. We found no evidence for effects of species and length on survival probability. Vitality class provides a useful qualitative prediction of survival probability within gear. These findings are relevant for the fisheries management of rays stocks in the North sea and Eastern Channel.
**Order of Authors:**	Edward Schram, Ph.D.
	Lennert van de Pol
	Katinka Bleeker
	Pieke Molenaar
	Allard Van Mens
	Karolina Molla Gazi
	Pim van Dalen
	Wouter Suykerbuyk
	Batsleer Jurgen
	Suzanne Cornelisse
**Additional Information:**	
**Question**	**Response**
**Financial Disclosure**<br><br>Enter a financial disclosure statement that describes the sources of funding for the	Yes

Powered by Editorial Manager® and ProduXion Manager® from Aries Systems Corporation

work included in this submission. Review the submission guidelines for detailed requirements. View published research articles from *PLOS ONE* for specific examples.<br><br>This statement is required for submission and **will appear in the published article** if the submission is accepted. Please make sure it is accurate.<br><br>**Funded studies**<br>Enter a statement with the following details:<br>• Initials of the authors who received each award<br>• Grant numbers awarded to each author<br>• The full name of each funder<br>• URL of each funder website<br>• Did the sponsors or funders play any role in the study design, data collection and analysis, decision to publish, or preparation of the manuscript?<br><br>Did you receive funding for this work?	
Please add funding details.<br>as follow-up to "**Financial Disclosure**<br><br>Enter a financial disclosure statement that describes the sources of funding for the work included in this submission. Review the submission guidelines for detailed requirements. View published research articles from *PLOS ONE* for specific examples.<br><br>This statement is required for submission and **will appear in the published article** if the submission is accepted. Please make sure it is accurate.	European Union, European Maritime and Fisheries Fund (EMFF)

**Funded studies**<br>Enter a statement with the following details:<br>• Initials of the authors who received each award<br>• Grant numbers awarded to each author<br>• The full name of each funder<br>• URL of each funder website<br>• Did the sponsors or funders play any role in the study design, data collection and analysis, decision to publish, or preparation of the manuscript?<br><br>Did you receive funding for this work?"	
Please select the country of your main research funder (please select carefully as in some cases this is used in fee calculation).<br>as follow-up to "**Financial Disclosure**<br><br>Enter a financial disclosure statement that describes the sources of funding for the work included in this submission. Review the submission guidelines for detailed requirements. View published research articles from *PLOS ONE* for specific examples.<br><br>This statement is required for submission and **will appear in the published article** if the submission is accepted. Please make sure it is accurate.<br><br>**Funded studies**<br>Enter a statement with the following details:<br>• Initials of the authors who received each award<br>• Grant numbers awarded to each author<br>• The full name of each funder<br>• URL of each funder website<br>• Did the sponsors or funders play any role in the study design, data collection and analysis, decision to publish, or preparation of the manuscript?<br><br>Did you receive funding for this work?"	NETHERLANDS - NL
**Competing Interests**	The authors have declared that no competing interests exist.

Use the instructions below to enter a competing interest statement for this submission. On behalf of all authors, disclose any competing interests that could be perceived to bias this work—acknowledging all financial support and any other relevant financial or non-financial competing interests.<br><br>This statement is required for submission and **will appear in the published article** if the submission is accepted. Please make sure it is accurate and that any funding sources listed in your Funding Information later in the submission form are also declared in your Financial Disclosure statement.<br><br>View published research articles from *PLOS ONE* for specific examples.<br><br>**NO authors have competing interests**<br><br>Enter: *The authors have declared that no competing interests exist.*<br><br>**Authors with competing interests**<br><br>Enter competing interest details beginning with this statement:<br><br>*I have read the journal's policy and the authors of this manuscript have the following competing interests: [insert competing interests here]*<br><br>* typeset	
**Ethics Statement**<br><br>Enter an ethics statement for this submission. This statement is required if the study involved:<br><br>• Human participants<br>• Human specimens or tissue<br>• Vertebrate animals or cephalopods<br>• Vertebrate embryos or tissues	Animal Research.<br><br>The treatment of the fish was in accordance with the Dutch animal experimentation act, as approved by the Centrale Commissie Dierproeven (CCD, Central Commission Animal Experiments) and evaluated by the Dier Ethische Commissie (DEC, Animal Ethical Commission) and the Instantie voor Dierenwelzijn van Wageningen Research (Commission for Animal Welfare).<br><br>Permit numbers are 2017.D-0012.002, 2018.D-0002.004 and 2021.D-0007.001.

• Field research<br><br>Write "N/A" if the submission does not require an ethics statement.<br><br>General guidance is provided below. Consult the submission guidelines for detailed instructions. **Make sure that all information entered here is included in the Methods section of the manuscript.**	

Powered by Editorial Manager® and ProduXion Manager® from Aries Systems Corporation

**Format for specific study types**<br><br>**Human Subject Research (involving human participants and/or tissue)**<br>• Give the name of the institutional review board or ethics committee that approved the study<br>• Include the approval number and/or a statement indicating approval of this research<br>• Indicate the form of consent obtained (written/oral) or the reason that consent was not obtained (e.g. the data were analyzed anonymously)<br><br>**Animal Research (involving vertebrate animals, embryos or tissues)**<br>• Provide the name of the Institutional Animal Care and Use Committee (IACUC) or other relevant ethics board that reviewed the study protocol, and indicate whether they approved this research or granted a formal waiver of ethical approval<br>• Include an approval number if one was obtained<br>• If the study involved *non-human primates*, add *additional details* about animal welfare and steps taken to ameliorate suffering<br>• If anesthesia, euthanasia, or any kind of animal sacrifice is part of the study, include briefly which substances and/or methods were applied<br><br>**Field Research**<br><br>Include the following details if this study involves the collection of plant, animal, or other materials from a natural setting:<br>• Field permit number<br>• Name of the institution or relevant body that granted permission	
**Data Availability**<br><br>Authors are required to make all data underlying the findings described fully available, without restriction, and from the time of publication. PLOS allows rare exceptions to address legal and ethical concerns. See the PLOS Data Policy and FAQ for detailed information.	Yes - all data are fully available without restriction

A Data Availability Statement describing where the data can be found is required at submission. Your answers to this question constitute the Data Availability Statement and **will be published in the article**, if accepted.<br><br>**Important:** Stating 'data available on request from the author' is not sufficient. If your data are only available upon request, select 'No' for the first question and explain your exceptional situation in the text box.<br><br>Do the authors confirm that all data underlying the findings described in their manuscript are fully available without restriction?	
**Describe where the data may be found in full sentences. If you are copying our sample text, replace any instances of XXX with the appropriate details.**<br><br>• If the data are **held or will be held in a public repository**, include URLs, accession numbers or DOIs. If this information will only be available after acceptance, indicate this by ticking the box below. For example: *All XXX files are available from the XXX database (accession number(s) XXX, XXX.)*.<br>• If the data are all contained **within the manuscript and/or Supporting Information files**, enter the following: *All relevant data are within the manuscript and its Supporting Information files.*<br>• If neither of these applies but you are able to provide **details of access elsewhere**, with or without limitations, please do so. For example:<br><br>*Data cannot be shared publicly because of [XXX]. Data are available from the XXX Institutional Data Access / Ethics Committee (contact via XXX) for researchers who meet the criteria for access to confidential data.*<br><br>*The data underlying the results presented in the study are available from (include the name of the third party*	In case our manuscript is accepted for publication, a Supporting Information file containing all data used for this study will be included to the revised submission.

and contact information or URL).<br>• This text is appropriate if the data are owned by a third party and authors do not have permission to share the data.<br><br>* typeset	
Additional data availability information:	

Powered by Editorial Manager® and ProduXion Manager® from Aries Systems Corporation

Manuscript Click here to access/download;Manuscript;Manuscript ray discards survivalPLoSOne.docx

Survival probabilities of thornback and spotted rays discarded by tickler chain beam trawl, pulse beam trawl and flyshoot fisheries

Edward Schram*, Lennert van de Pol, Katinka Bleeker, Pieke Molenaar, Allard van Mens, Karolina Molla Gazi, Suzanne Cornelisse, Pim van Dalen, Wouter Suykerbuyk, Jurgen Batsleer

Wageningen Marine Research, Wageningen University & Research, P.O. Box 67, 1970 AB, IJmuiden, The Netherlands.

* Corresponding author

E-mail: Edward.schram@wur.nl

Keywords: Discards survival, rays, North Sea, landing obligation, beam trawling, flyshoot

Abstract

To measure discards survival probabilities of thornback (*Raja clavata)* and spotted ray (*Raja montagui*) we conducted five trips with a tickler chain beam trawler during which 183 thornback and 137 spotted rays were sampled, nine trips with pulse beam trawlers during which 94 thornback rays were sampled and four trips with a flyshooter during which 137 thornback and 24 spotted rays were sampled. Survival probabilities were measured by captive observation for 15 to 25 days post catch. All fishery operations were conducted in the southern North Sea (ICES division 27.4.c) and in the Eastern English Channel (ICES division 27.7.d) according to the regular commercial practices of the fishing vessels. Trips were spread out over the seasons to account for the effect of variable environmental and fishing conditions on discards survival. Operational and environmental conditions during sea trips were recorded. For beam trawling survival probabilities (95% CI) were 50% (43-57) and 44% (37-54) for thornback and spotted ray. For pulse trawling survival probability of thornback was 54% (40-65). For flyshooting survival

probabilities were higher: 80% (73-87) for thornback and 75% (60-94) for spotted ray. Survival probabilities were significantly affected by gear, catch processing time, water temperature, wave height and the interaction between water temperature and wave height. We found no evidence for effects of species and length on survival probability. Vitality class provides a useful qualitative prediction of survival probability within gear. These findings are relevant for the fisheries management of rays stocks in the North sea and Eastern Channel.

Introduction

Skates and rays play important roles as predators in the marine ecosystem [1]. They are long-lived, slow growing, reach sexual maturity at relatively old age, and produce a small number of offspring per year. These specific biological characteristics make skates and rays vulnerable to overexploitation and changes in essential habitats, especially spawning and nursery areas [2,3,4,5]. The decline of ray populations in the North Sea, especially in the last century, is generally attributed to long-term intensive fishing as well as large-scale coastal infrastructure and pollution [5,6,7]. Fisheries independent data show an increase in abundance for some species, e.g. thornback ray (*Raja clavata*) and blonde ray (*Raja brachyura*) since 2010 [8].

In the North Sea, rays are mainly caught as by-catch in mixed demersal fisheries for flatfish [9,10] and managed under a single, common Total Allowable Catch (TAC) [11]. Commercially the most important species are thornback ray (*Raja clavata*), blonde ray (*Raja brachyura*) and spotted ray (*Raja montagui*). Landings of starry ray (*Amblyraja radiata*) and common skate (*Dipturus* sp.) are prohibited. Next to the TAC, local management measures such as seasonal closures and gear restrictions and minimum landing sizes (MLS) also regulate exploitation of these species. Landings are currently restricted to rays larger than 45 to 55 cm, varying by country, and by landing limits (kg) per trip to prevent overexploitation. Rays below the MLS and surplus catches of rays are discarded. Since 2019, the practise of discarding has been restricted by the implementation of a landing obligation (LO) under the Common Fisheries Policy (article 15 of the EU regulation No 1380/2013) [12], which requires fishers to land their entire catch of species under TAC management, including rays. Because landing results in 100% mortality, species with a high survival probability when discarded are eligible for exemptions to the landing obligation.

Fisheries have a clear interest in these exemptions because it mitigates the risk of early depletion of quota and subsequent closures of fisheries when catches of these so called ‘choke species’ cannot be avoided. Because of the relatively small TAC, rays are particularly prone to become choke species. Marine nature

conservationists also support exemptions to the LO to reduce unnecessary fishery mortality among rays, especially because their survival probability is expected to be relatively high [13].

Among Dutch demersal fisheries, beam trawl and flyshoot fisheries are quantitatively the main contributors to both ray discarding and landings, with thornback and spotted ray as main species [14]. Discards survival probability measurements are thus most relevant for these metiers and species. Previous studies on ray discards survival in North Sea beam trawl fisheries report short-term survival (60 – 120 h post catch monitoring, e.g. [15,16,17] and this may be too short to determine the fate of the sampled fish [18]. Van Bogaert et al. [20] monitored survival up to 21 days post catch for a limited number of thornback rays (n=21). Survival probabilities of rays discarded by flyshoot fisheries have not been measured.

The objectives of this study were to measure survival probabilities of rays discarded by tickler chain and pulse beam trawl fisheries and flyshoot fisheries. We conducted 18 trips spread out over the seasons during which in total 397 thornback rays and 161 spotted rays were sampled from commercial catches. Survival probabilities were determined by captive observations of the sampled rays for 15 to 25 days post catch.

Materials and methods

Ethics statements

The treatment of the fish was in accordance with the Dutch animal experimentation act, as approved by ethical committees (Experiments, 2017.D-0012.002, 2018.D-0002.004 and 2021.D-0007.001). The methodology was in accordance with the guidelines for discards survival studies developed by the Workshop on Methods for Estimating Discard Survival (WKMEDS) of the International Council for the Exploration of the Sea (ICES) [18]. Release of surviving rays to the Eastern Scheldt after the completion of experiments was in accordance with IUCN (International Union for Conservation of Nature) Guidelines for reintroductions and other conservation translocations [19].

Experiments

Trips

Test-fish were collected during eighteen regular commercial fishing trips on commercial pulse trawlers (TBB_pulse, 9 trips, Table 1, S1), tickler chain beam trawlers (TBB, 5 trips, Table 1, S2) and a commercial flyshooter (SSC, 4 trips, Table 1, S3) between 2017 and 2023. All trips lasted between four and five days, comparable to commercial trip lengths. All fishery operations were conducted in the southern North Sea and the English Channel according to commercial practices of the fishing vessels. Trips were spread out over the year (Table 2) to account for the potential effect of variable environmental and fishing conditions on discards survival [21,22]. Operational and environmental conditions during sea trips were recorded for each haul on trawl lists by the skippers (Table 2).

Table 1 Fishing vessels used.

Vessel	1	2	3	4	5
Gear	TBB-pulse	TBB-pulse	TBB-pulse	TBB	SSC
Engine power (Kw)	1471	1430	1470	1888	680
Tonnage (GT)	426	366	494	494	340
Length (m)	41	39	42	42	31
Fishing speed (kn)	4.8	4.8	4.9	6.1	n.a.

Table 2: Conditions during the sea trips.

Trip	Métier	Vessel	Year	Month	Week	Mean water temp. (°C)	Wind speed range (Bft)	Mean wave height (m)	Mean catch processing (min)	Mean haul duration (min)	Mean fishing depth (m)
1	TBB_pulse	1	2017	May	18	9-12	2-5	0.5-2.0	30	110-135	18-28
2	TBB_pulse	2	2017	May	21	12-13	0-4	0.2-0.5	24	120	30-50
3	TBB_pulse	3	2017	June	24	14-15	1-5	0.1-1.5	20	110-125	22-24
4	TBB_pulse	3	2017	July	28	16-17	1-6	0.1-1.0	23	110-120	25-40
5	TBB_pulse	1	2017	Sept	36	18	4-5	0.5-1.5	26	120-145	26-37
6	TBB_pulse	3	2017	Oct	44	13-15	3-5	0.5-2.0	20	110-130	27-34
7	TBB_pulse	2	2017	Dec	49	11-12	3-5	1.0-2.0	34	120	35-50
8	TBB_pulse	1	2018	Jan	4	6-7	5-6	0.5-2.6	33	120	28-39
9	TBB_pulse	2	2018	Feb	8	7-8	2-5	0.5-1.5	25	110-120	22-52
10	TBB	4	2021	Oct	41	15.9	3-4	1.14	17	126	27
11	SSC	5	2021	Nov	47	13.5	2-5	1.1	44	88	32
12	TBB	4	2022	Jan	3	8.7	1-4	1.7	28	126	28
13	SSC	5	2022	Mar	13	10.0	2-4	1.3	24	69	45
14	TBB	4	2022	May	19	11.3	1-4	0.7	24	124	27
15	SSC	5	2022	Jun	24	16.0	1-4	0.5	26	67	38
16	TBB	4	2022	Jun	26	15.6	1-3		23	124	27
17	SSC	5	2022	Aug	33	20.2	0-4	1.0	24	67	37
18	TBB	4	2022	Sep	38	18.1	1-6	0.9	24	121	26

Test-fish collection

In total 558 test-fish were sampled with thornback rays ranging from 17 to 92 cm and spotted rays ranging from 24 to 63 cm total length (TL). All rays were sampled during the semi-automatic catch-sorting process which is common in these fisheries. In this process, catches were discharged from the cod-end(s) into one (flyshoot) or two (pulse and beam trawl) hoppers. From the hoppers, the catches were flushed into a central pit from which the catch was transported by a conveyer belt onto the sorting belt from which marketable fish were manually collected belt by crew members. At the end of the sorting belt, the remaining unwanted catch, including fish with no commercial value and undersized fish, dropped into a gutter with a water supply that discharged the catch back into the sea.

Trips with pulse trawlers were dedicated to discards survival measurements for a variety of species including thornback ray. We aimed for balanced sampling of catch processing of three rays per haul but this was occasionally hampered by availability of rays in the catches. The total number of sampled rays per pulse trawl trip ranged from nine to fourteen specimens from three to seven hauls per trip.

For the beam trawl and flyshoot trips, that were fully dedicated to discards survival measurements of rays, the total of 40 rays < 65 cm TL and 6 > 65 cm TL that could be sampled per trip was determined by the housing capacity in the on-board monitoring units (see below). We aimed to sample 20 thornback and 20 spotted rays smaller than 65 cm TL each trip by sampling five rays from four hauls for both species. Each sampled haul we aimed to sample the first two and last three rays per species that appeared on the sorting belt to account for the potential effect of time spent in catch processing on survival probability. Low numbers of rays in some of the catches incidentally forced us to deviate from this sampling scheme. Occasionally, rays were sampled from the mid part of catch processing to obtain the desired sample size per haul. Each beam trawl and flyshoot trip large thornback rays (> 65 cm TL) were opportunistically sampled according to their availability in catches. Spotted rays > 65 cm TL were not available in any of the sampled hauls. Rays that were dead when sampled or died at sea, and housing of two small rays (< 25 cm) in one tank were used as opportunities to sample more rays per trip. For each sampled haul the start and end time of catch sorting were recorded to obtain the approximate sampling time of individual rays.

Control fish

During all trips, control fish of the same species as the test-fish (Trips 1 to 9, n = 2; Trips 10 to 18 n=4 per species) were used to separate potential effects of the experimental procedures on mortality from fisheries-induced mortality. At the start of a trip, control fish were stored on deck in aerated 600L tanks. At sea the tank water was regularly renewed with surface seawater. Control fish were exposed to the exact same experimental procedures throughout the experiments as test-fish as of the moment of test-fish collection from the sorting belt. Control fish were obtained by collecting the least damaged rays from the catches of a research vessel and by using test-fish from previous trips as control fish. Prior to their use in experimental trips, control fish were stored in tanks placed in a climate-controlled room for at least three weeks. During this period, fisheries-induced mortality levelled out while surviving fish could recover from injuries and regain good condition. During storage, fish were fed daily with dead, uncooked brown shrimps (*Crangon crangon*) and pieces of herring (*Clupea harengus*) and Atlantic mackerel (*Scomber scombrus*) to visually observed satiation. Only fish in visually observed good condition, well-fed and without visible injuries, were used as control fish.

Condition assessment and monitoring of survival

After sampling from catches, fish were temporarily stored in 105L holding containers filled with seawater. Seawater in holding containers was regularly renewed to maintain dissolved oxygen levels during storage. Once sampling of a haul had been completed, fish were sequentially taken from the holding containers for condition assessment, to measure total length (TL: in cm below), to determine their sex and for tagging. Fish condition of each individual fish was determined and scored A to D by scoring reflex impairment and damages, using a methodology developed for flat fish [21] that was adapted to rays after [20] (Table 3). Fish were tagged with Trovan Unique glass transponders (type ID100) to allow for identification of individuals. Transponders were injected subcutaneously at the base of the tail using an IID100E injector. Upon completion of their initial assessment, rays were individually placed in 24 L tanks (TL < 65 cm) or 84 L tanks (TL > 65 cm). Occasionally two small specimens (TL < 25 cm) were placed in a single 24 L tank that was split into two equally sized compartments by a perforated plexiglass sheet. Fish that were considered dead when sampled (no spiracle movement for more than 15 seconds) were recorded as dead at time zero and not placed in tanks. Survival monitoring started at sea and continued in the laboratory. All tanks were inspected for mortalities every 12 hours on board and every 24 hours in the laboratory. Dead fish were detected by visual confirmation of the absence of spiracle movement, immediately removed from their tanks and identified by their PIT tags. The date and time at which a ray was found dead was recorded. Lethargic fish were not removed as for their potential recovery and to obtain actual survival time. Dissolved oxygen concentration and water temperature were measured (Hach Lange Multimeter). Water flows to individual tanks were increased if oxygen saturation was below 80%. All experiments were terminated after 14 (trip 1 to 9) or 21 (trip 10 to 18) days of survival monitoring in the laboratory. Depending on the day of sampling at sea, this resulted in post catch monitoring periods for surviving individual rays ranging between 15 and 18 days for trip 1 to 9 and 22 and 25 days for trip 10 to 18. Upon termination of the experiment all surviving rays were netted from the tanks and identified by their PIT tags to record species, total length (TL: in cm below) and sex. Surviving rays were either released to the Eastern Scheldt or kept in captivity for use as control fish in upcoming survival experiments.

Table 3: Description of criteria to score condition and determine vitality class of rays (methodology for flat fish by Van der Reijden et al. [21] modified for rays after Van Bogaert et al. [20].

**Vitality class**	Description
A	Fish lively, no visible or very minor external damages.
B	Fish less lively, minor scratches or discolorations on up to 20% of the dorsal skin surface area, some haemorrhaging on the ventral side, no or minor bruises on the ventral side.
C	Fish lethargic, intermediate scratches or discolorations on up to 50% of the dorsal skin surface area, several haemorrhaging and/or bruising on the ventral side.
D	Fish lethargic or dead, major scratches or discolorations on the dorsal skin surface area, significant haemorrhaging and/or bruising on the ventral side.
**External damage scores**	Description (scoring: 1 = present; 0 = absent)
Wings	Wings are damaged or split.
Dorsal side	Damage to skin surface, scratches or discolorations at dorsal pigmented body surface.
Hypodermic haemorrhages	Superficial hypodermic haemorrhage on the ventral white body surface
Hypodermic bruises	Hypodermic purple-reddish bruise on the ventral white body surface
Intestines	Intestines are protruding or are visible through damaged body tissue of the fish.
Wound	A wound such that flesh is visible anywhere on the body including torn off thorns.
**Reflex impairment scores**	Description (1 = impaired; no (clear) response within 5 s of observation; 0 = unimpaired; obvious response within 5 s).
Wings	Ray is held out of the water, dorsal side up with one hand supporting the body at the head of the ray and the other hand supporting the body at the base of the tail. The ray actively flaps its pectoral fins (wings).
Eye retraction	While out of the water the ray is gently tapped on the head just behind the eyes with a blunt probe. The ray actively retracts its eyes.
Tail grab	While in the water resting on the tank bottom the ray is gently held by the tail. When the tail is gently pulled backwards, the ray struggles free and swims away (or attempts to do this).
Spiracles	While in the water resting on the tank bottom the movement of the spiracles is observed.

Experimental facilities

Two different types of custom-built monitoring units were installed on board the fishing vessels to house test-fish and control fish. Type 1 units consisted of a stainless-steel framework holding 16 polyethylene 24L tanks (60 cm L x 40 cm W x 12 cm H) suitable for rays with a total length < 65 cm. The type 2 unit held six polyethylene 84 L tanks (80 cm L x 60 cm W x 17.5 cm H) to house six rays with a total length > 65 cm. During trips 1 to 9 (pulse trawl trips) four type 1 units were installed on board, resulting in a total capacity of 64 tanks of which nine to fourteen were available for rays while the other tanks were used to house flat fish species (not reported on here). Trips 10 to 18 (beam trawl and flyshoot trips) each employed three type 1 units and one type 2 units, resulting in a total capacity to house 48 rays < 65 cm TL and 6 rays > 65 cm TL. In all units, each tank was equipped with an individual water supply. A pump with a water intake approximately 2 meters below sea surface continuously supplied seawater to the tanks. Water flow rates were approximately two tank volumes per hour to maintain proper water quality. Tanks were covered with transparent lids to limit water loss by sloshing while allowing for visual inspection of fish. Upon return in port, the units were transported to the laboratory in a temperature controlled truck. Transport time ranged from one to four hours depending on the home port of the vessel. During transport each unit was placed inside a tank that was partly filled with seawater and equipped with a submerged pump to supply water to each fish tank in the unit. Fish tanks discharged their effluents in the tank in which the unit was placed, allowing for recirculation and aeration of the water. Upon arrival at the laboratory, fish were removed from their tanks and housed in a tank system placed in a temperature controlled room. Rays > 65 cm TL were housed in tanks with a bottom surface area of 2.5 m^2 at a maximum density of 4 fish per tank (1.6 m^2/fish). Rays < 65 cm TL were housed in tanks with a bottom surface area of 2 to 2.5 m^2 at a maximum density of 8 fish per tank (0.5 to 0.8 m^2/fish). Control fish were mixed with test fish. Different species were housed in separate tanks. All tanks were connected to a single water recirculation system with a biological filter. Water in the system was continuously renewed with filtered seawater from the Eastern Scheldt at a rate of 2 to 3 m^3/d. In the laboratory, all tanks were supplied with coarse sand as bottom substrate and fish were fed daily to visually observed satiation with uncooked brown shrimps (*Crangon crangon*) and pieces of herring (*Clupea harengus*) and Atlantic mackerel (*Scomber scombrus*).

Data analysis

Survival curves

To visualise and analyse the survival of the sampled fish, Kaplan-Meier survival curves [23] were fitted and 95% Confidence Intervals were computed using *survfit* function from the R-package *survival*. Survival curves and their 95% Confidence Intervals were created for control fish vs. test-fish by gears and species and for test-fish by vitality class. Fish that died due to unnatural causes, mainly by jumping from the containment tank, were censored: this means that rather than being registered as dead, their experiment was said to have been terminated at the time of death.

Statistical tests survival

To test if differences in survival were significant between species, gears and vitality classes, the functions *survdiff* and *pairwise_survdiff* from the *survival* package were used, which take the times until death (or censoring) and a categorical group and computes a test statistic based on the log-rank test [24]. The log-rank test statistic is calculated by comparing the observed and expected number of events in each group at various time points throughout the study period. It measures the discrepancy between the observed and expected events under the assumption that the survival experiences for all groups are identical. This was done to detect differences in discards survival probabilities of thornback and spotted ray between gears and vitality classes. Effects were considered significant when $p < 0.05$.

Predictors of survival

Generalized Linear Mixed-Effect Models (GLMMs) were computed to identify the combination of variables that best explained the variation in discard survival, using the *glmer* function from the *lme4* package in R. In these models, the haul from which fish were sampled (incorporating the variables vessel and trip) was included as random effect. Models of all combinations of the explaining variables gear, species, length, catch processing time, sea surface temperature, depth and wave height, including all possible interactions were computed using the *dredge* function from the *MuMIn* package in R. Haul duration was not considered because variation was low and because the haul process is different between (pulse) beam trawling and flyshooting, meaning haul duration cannot be considered analogous between the different fishing methods. Wind speed was not considered because of its strong correlation to wave height. Sediment type was not considered because the majority of observations occurred on the same sediment type (sandy). The numerical variables were normalized to increase the performance of the models. The model with the lowest Akaike Information Criterion (AIC) value was considered the best fit.

Vitality class as predictor of survival

To investigate the effectiveness of vitality class a predictor of discard survival, GLMMs were computed employing the selected combination of variables that best explained the variation in discards survival, as well as vitality class. Vitality class was considered a generic predictor of survival in case it was the only variable selected in the GLMM with the lowest AIC.

The presence of observer bias [24] in the vitality classification was investigated by computing a Cumulative Link Mixed Model (CLMM) with vitality class as dependent variable and observer as explaining variable. Using the *drop1* function from the *stats* package in R, we performed a hypothesis test based on the chi-squared distribution to assess the significance of dropping the observer variable from the model. Observer bias was present in case the model with observer as explaining variable was significantly better at predicting vitality class than the null model ($p < 0.05$).

Results

Overall discards survival probabilities

For all gear types and species the overall survival probability estimates were based on trips spread out over the year and conducted under variable environmental conditions (Table 2). Overall survival probability estimates for thornback ray differed among gears ($p<0.0001$, Table 4). The discards survival probability observed for thornback ray in flyshoot fisheries was higher than the probabilities observed for beam trawl and pulse trawl fisheries ($p < 0.0001$). No difference in survival probability of thornback ray was detected between beam and pulse trawl fisheries ($p = 0.48$). Overall survival probability estimates for spotted ray differed between flyshoot and beam trawl fisheries ($p = 0.01$, Table 4). For pulse trawl fisheries no spotted rays were sampled.

For both flyshoot and beam trawl fisheries most of the mortality among spotted rays occurred within the first two to five days of the experiment and levelled out thereafter (Fig. 1b and 1e). Mortality among thornback rays occurred up to approximately day 20 for beam trawl fisheries (Fig. 1a), up to day 15 for pulse trawl fisheries (Fig. 1c) and up day 10 post catch for flyshoot fisheries (Fig. 1d) before levelling out. Direct mortality, i.e. dead at-vessel when sampled, just before the moment rays would have been discarded, was lowest for pulse trawl fisheries (Table 4). Only one out of a total of 86 control fish died in the experiments, resulting in control fish survival ranging from 94% for pulse trawling to 100% for the other two gears.

Table 4: Overall discards survival probability estimates (%) and their 95% confidence intervals, direct mortality and survival of control fish for spotted and thornback ray discarded by beam trawl, pulse trawl and flyshoot fisheries. Survival probability estimates within species and per gear with different letters in superscript are significantly different. Note that no comparisons between species were made.

Species	Gear	Survival probability (95% CI)	n	Direct mortality	Survival of control fish	n
Thornback ray	Beam trawl	50% (43-57)[a]	183	11.8%	100%	21
	Pulse trawl	54% (40-65)[a]	94	2.1%	94%	18
	Flyshoot	80% (73-87)[b]	120	4.0%	100%	16
	p-value	$p < 0.0001$				
Spotted ray	Beam trawl	44% (37-54)	137	11.3%	100%	19
	Flyshoot	75% (60-94)	24	2.5%	100%	12
	p-value	$p = 0.02$				

Fig. 1. Kaplan-Meier survival curves for thornback (a,c,d) and spotted ray (b,e) discarded by beam trawl (a, b), pulse trawl (c) and flyshoot fisheries (d, e). Curves are plotted for control fish and test-fish employing all fish sampled during five beam trawl trips, nine pulse trawl trips and and four flyshoot trips. Drawn lines indicate mean survival (percentage over time), with shaded areas indicating 95% confidence intervals. Dots indicate the end of the monitoring time for individual fish that were alive at the end of the experiments.

Predictors of survival

Survival was best explained by the GLMM including the variables gear, water temperature, catch processing time, wave height and the interaction between wave height and water temperature. Variables fishing depth, length and species were not selected. Table 5 presents the estimates and their significance of the fixed effects. All selected variables have a significant effect on discards survival probability except for the gear effect of pulse trawling, i.e. survival probability does not differ between pulse and beam trawling. Figure 2 gives the probability distributions with 95% confidence intervals for the effects of the selected variables on survival probability at each value of the fixed effect with the other variables set to the mean value (or mode for non-numeric variables). Survival probability differed among gears (Fig. 2a) with higher survival for rays discarded by flyshoot fisheries ($p < 0.001$) but no difference between pulse and beam trawl fisheries ($p=0.58$). Survival probability decreases with catch processing time (Fig. 2c, p

= 0.024) and to a lesser extent with wave height (Fig. 2d, p = 0.02) and water temperature (Fig. 2b, p = 0.027). The effect of water temperature depends on wave height (Fig. 2e, p = 0.022).

Fig. 2 Predicted probabilities of discards survival for the variables in the GLMM with the lowest AIC value. Variables selected in the model were a) gear, b) water temperature, c) catch processing time, d) wave height and e) the interaction between water temperature and wave height. The graphs represent the probability of survival at each value of the fixed effect with the other variables set to the mean value (or mode for non-numeric variables).

Table 5 Estimates for the fixed effects and their significance in the Generalized Linear Mixed-Effect Model explaining the survival probability of thornback and spotted ray discarded by beam trawl, pulse trawl and flyshoot fisheries. Gear effects are relative to beam trawl fisheries. Significant fixed effects are marked with *.

Fixed effects:	Estimate	Std. Error	z value	Pr(>|z|)
Intercept	2.1118	0.8303	2.543	0.011 *
Gear Flyshoot	2.0018	0.3875	5.166	<0.001*
Gear Pulse trawl	0.1991	0.3627	0.549	0.58
Wave height	-4.5603	1.9667	-2.319	0.02 *
Water temperature	-2.4954	1.1306	-2.207	0.027 *
Catch processing time	2.7659	-1.2236	-2.260	0.024 *
Water temperature*Wave height	6.1802	2.6887	2.299	0.022 *

Vitality class as predictor of survival

The condition in which rays were landed on deck was expressed by vitality classes A to D. Vitality class had a significant effect on discards survival probability (Table 6) and for all species-gear combinations survival probability declined with deteriorating condition. In all cases survival probability was highest for vitality class A and lowest for vitality class D, but not all observed survival probabilities differed among vitality classes (Table 6). GLMMs employing the previously selected variables that best explain survival probability (gear, catch processing time, water temperature, wave height) plus vitality class were computed to investigate to what extend vitality class is a generic predictor of survival probability. Survival was best explained (lowest AIC) by a model that included vitality class, gear, water temperature and the interactions between gear and water temperature and between temperature and vitality. Apparently vitality class incorporates the effects of catch processing time and wave height on survival as

these variable were no longer selected. Kaplan-Meier survival curves by vitality class reveal distinct differences in survival between vitality classes for beam and pulse trawl fisheries (Fig. 3a) while for flyshoot fisheries vitality classes A, B and C show more similar survival curves (Fig. 3b). This, combined with the selection of the gear and water temperature in the model, reveals that vitality class is not a generic predictor of ray discards survival. No observer bias in vitality classification was detected; the CLMM with observer as explaining variable was not significantly better than the null model (p=0.44).

Fig. 3 Kaplan-Meier survival curves for test-fish of vitality classes A to D discarded by beam and pulse trawl (a) and flyshoot fisheries (b) combining the observations for thornback and spotted ray. Note that vitality class D includes fish that were dead at the start of the experiment. Drawn lines indicate mean survival percentage over time, with shaded areas indicating 95% confidence limits.

Table 6. Survival probability estimates (%) and their 95% confidence intervals per vitality class of spotted and thornback rays discarded by beam trawl, flyshoot and pulse trawl fisheries. The letters in superscript refer to the vitality classes from which the survival probability is significantly different (within gear and species).

Species	Gear	A	B	C	D	p-value
Spotted ray	Beam trawl	100% (100-100)^{C,D}	74% (64-86)^{C,D}	19% (8-41)^{A,B,D}	5% (1-19)^{A,B,C}	p << 0.001
	Flyshoot	100% (100-100)	73% (54-100)^{D}	80% (52-100)	0%^{B}	p << 0.001
Thornback ray	Beam trawl	97% (90-100)^{D}	70%(60-81)^{C,D}	50% (37-66)^{B,D}	12% (6-25)^{A,B,C}	p << 0.001
	Flyshoot	97% (90-100)^{D}	90%(83-98)^{D}	79% (65-95)^{D}	39% (22-69)^{A,B,C}	p << 0.001
	Pulse trawl	90% (80-100)^{C,D}	67% (53-84) ^{C,D}	44% (30-65)^{A,B,D}	8% (1-54)^{A,B,C}	p << 0.001

Discussion

The main objective of this study was to provide representative estimates for the survival probabilities of rays discarded by beam trawl, pulse trawl and flyshoot fisheries. To this end we conducted 18 trips spread out over the seasons during which in total 397 thornback rays and 161 spotted rays were sampled from commercial catches. Sampled rays were housed in tanks to monitor their survival over time. The artificial conditions and fish handling associated to captive observation experiments may cause additional mortality through additional stress [18] and thus underestimate actual discards survival. In

our experiment however, we consider all mortality among sampled rays to be fisheries induced because all but one control fish, employed to separate mortality caused by the experimental procedures from fisheries-induced mortality, survived. Captive observation may also result in overestimation of actual discards survival because it excludes the contribution of predation to discards mortality [26]. Grey seals (*Halichoerus grypus*) predate on rays [27,28] and show feeding activity associated to fisheries [29]. Although there are to the best of our knowledge no records of fisheries associated ray predation by grey seals, we cannot rule out that it occurs and affects discards survival probability. In addition, rays recovering on the seafloor may be prone to mortal injury by bottom scavengers [30]. Because of these unknown levels of predation related mortality, the discards survival probability estimates reported here should be considered as maximum values.

To the best of our knowledge this study presents for the first time discard survival probabilities for rays discarded by flyshoot fisheries. Consequently, there are no previous studies to which we can compare our results. There are only a few other studies on ray discards survival in North Sea beam trawl fisheries. Apart from the study by Van Bogaert et al. [20], these other studies report short-term survival. Short-term survival (80h) of 72% was observed for unspecified ray species (given the sampling area probably at least partly thornback and spotted rays) sampled from catches of eurocutters with chain mat beam trawls [17]. Kaiser and Spencer [15] report 59% survival after 5 days of monitoring for cuckoo ray (*Leucoraja naevus*) caught by a 4 m beam trawl towed for 0.5 h. Enever et al. [16] report 59% short term (60h) survival for thornback rays caught in commercial beam trawl tows in the Bristol Channel. Because captive observation requires a sufficiently long monitoring period to determine the fate of the sampled fish [18] and our survival curves reveal that, especially for thornback ray, monitoring for at least 15 days post catch is required for mortality to level out, it is highly likely that these short-term studies report slightly overestimated survival probabilities. Note that we monitored survival for 15 days in our first ray discards survival experiments for pulse trawl fisheries and decided based on that first experiment to extend the monitoring period to 21 days in our later experiments for flyshoot and beam trawl fisheries to be on the safe side.
Mortality among fish caught and discarded by commercial fisheries is caused by failure to recover from fisheries induced stressors such as hypoxia, injury, exhaustion, barotrauma and predation to which these fish are exposed during capture, handling and release [31,32]. The effects of gear, catch processing time, water temperature and wave height on ray discards survival that we observed, can therefore be attributed to the effects of these variables on the severity of fisheries induced stressors.

The higher survival that we observed in flyshoot fisheries, can be explained by the shorter time fish are retained in the cod-end in this fisheries (maximum 20 minutes) compared to pulse and beam trawling (up to 120 minutes) and smaller amounts of benthic organisms and debris in the catches of flyshoot fisheries. Both result in lower mechanical impact, less injuries and exhaustion compared to beam and pulse trawling. Surprisingly we did not detect a difference in survival probability between pulse and beam trawl fisheries. We predicted a lower survival in beam trawling because of the higher towing speed of tickler chain beam trawls (6-7 kn.) and generally larger catch volumes with more benthic organisms and debris compared to pulse beam trawls (~ 5 kn.). Higher towing speeds may lead to lower discards survival through faster exhaustion, more injuries due to more severe collisions with the net, other fish and debris, more dense crowding and increased compression of fish in the cod-end.
It should be noted that within gear variation in discard survival may exist between individual vessels due to differences in fishing and catch processing procedures. Unless an experiment consists of a large number of trips, it will always be very difficult to disentangle vessel effects from effects of inevitably varying sea conditions between consecutive trips with different vessels. Differences in fishing and catch processing procedures are probably much larger between gears than among vessels that use the same gear. Therefore, we attribute the difference in discards survival probabilities observed between beam trawl and pulse trawl fisheries on the one hand and flyshoot fisheries on the other hand to a gear effect rather than a vessel effect. Also, we consider the survival probability estimates representative for the gears.

As discards survival probability declines with increased duration of hypoxic conditions [31], it is not surprising that survival probability declined with increasing catch processing time, i.e. increasing air exposure time. During air exposure, oxygen deficiency in tissues generally occurs due to collapsed gill lamellae which drastically reduces the fish' gas exchange capacity [31]. It should be noted however that effects of variables catch processing time, catch mass and haul duration are closely related: longer hauls lead to larger catches which lead to longer catch processing time. Each variable may affect discards survival by itself or by its effect on another variable. For example, decreasing survival with increasing haul duration, reported by e.g. [33], has been attributed to longer exposure to the mechanical impacts, i.e. compression [34] imposed on fish in the trawl as well as the increased catch mass resulting from longer tows [16,35]. Increased catch mass itself leads to denser crowding which exacerbates bruising, crushing and constriction injuries as fish are pushed against the cod-end mesh, other biota and debris [36]. This collinearity makes it is difficult to attribute effects on survival to individual variables. Unfortunately, we lack both catch mass data (not measured) and useful haul duration data (no contrast due to sampling from regular, unmanipulated commercial fisheries) to explore their effects on survival

probability of discarded rays. We therefore cannot exclude that effects of catch mass contributed to the observed effect of catch processing time on survival probability.

Provided that air exposure during catch processing contributed to ray discard mortality, survival may be increased by discharging catches in water filled hoppers instead of dry hoppers. Well designed and managed water filled hoppers in which the fish do not deplete dissolved oxygen levels, may reduce the impact of hypoxia because it shortens air exposure time during catch processing. Plaice discards survival did not benefit from water filled hoppers because most of the plaice caught with pulse beam trawls were already lethally damaged in the trawl prior to being discharged in the hoppers. This also explains why survival of these plaice was unaffected by catch processing time [22]. In case of rays however, our observation that catch processing time does matter suggests that in contrast to plaice, ray discards survival may benefit from water filled hoppers.

Lower survival probability with increasing surface water temperature, as we found for discarded rays, has been previously reported for chondrichthyans [37] as well as teleost fish, e.g. [22,38]. This temperature effect can be attributed to an increase in physiological stress at higher temperatures and exposure to temperature change [31]. Whether the current temperature effect was caused by the higher temperature itself or exposure to temperature change, which is largest during summer months [39], is not clear. The temperature effect seems rather small at first. However, the potential effect of temperature on survival is revealed by the interaction between water temperature and wave height. When waves are low, water temperature has a much stronger negative effect on survival probability than when temperature is considered as a main effect with all other variables in the model are set at their average values. The high survival at low water temperature when seas are calm is strongly reduced as soon as wave height increases. This effect of sea state is probably caused by an increase in mechanical impact of the gear on the fish [31] overruling the temperature effect. There is no logical explanation for a positive effect of wave height on survival at higher water temperature and we consider this model result an artifact of the limitations of the GLMM. More complex models seem to be needed to describe this interaction at high water temperatures.

In contrast to Van Bogaert et al. [20] we found no significant difference in the survival probability between thornback and spotted ray. Numerically however and in agreement with Van Bogaert et al. [20] we observed the highest survival for thornback ray. Thornback ray's more rugged and spinulose external surface has been suggested to offer better physical protection to mechanical impacts of capture processes and thus higher survival chances when discarded compared to more smoother ray species such as blonde (*Raja brachyura*) and spotted ray [16].

We found no evidence for a length effect on survival probability. This is surprising because both Van Bogaert et al. [20] and Depestele et al. [17] report that smaller specimens have lower survival chances. We currently have no explanation why our findings deviate but it seems unlikely that it is caused by a lack of length variation in our data that cover a wide length range.

All test-fish were assigned a vitality class A, B, C or D based on individual condition assessments directly after sampling. Survival probabilities among test-fish grouped by vitality class differed across species and gears. Without exception vitality classes A and D always yield the highest and lowest discards survival probabilities. Vitality class thus proved to be a fairly good predictor for ray survival, similar to flatfish [24]. Also, it is clear that the condition in which the fish arrive on the sorting belt has a strong effect on their survival chances when discarded. Survival chances can thus be increased by measures that reduce deterioration of fish condition prior to their landing on deck. The effect of vitality class on survival was most pronounced for rays caught by beam and pulse trawl fisheries and less in flyshoot fisheries. Also, survival by vitality class was higher in flyshoot fisheries, which suggests that in beam and pulse trawl fisheries rays died due to injuries that were not picked up by the condition assessment we used. In addition, other variables next to vitality class were selected when computing GLMMs to predict survival from vitality class. We therefore doubt that vitality classes as we assigned them, are generic predictors of ray discards survival and that accurate prediction of survival based on our condition assessment is possible. We consequently did not attempt to construct a mathematical model that predicts of discards survival using vitality class and the underlying reflex impairment and damage scores. For the moment, assigning vitality classes seems a useful tool for qualitative assessments of survival probabilities, e.g. to gain first insight in effects of gear modifications within gears on survival probabilities.

Conclusions

We consider the overall discards survival probabilities that we report in this study for thornback and spotted ray as maximum values that are representative for year-round beam trawl, pulse trawl and flyshoot fisheries. Discard survival of thornback and spotted ray is affected by gear, water temperature, sea state, catch processing time and the condition in which rays are landed on deck. Vitality class provides a useful qualitative prediction of survival probability within gear. To further increase survival probability, it is recommended to keep catch processing time as short as possible and to focus on gear modifications that reduce stressors inflicted upon fish during the catch and hauling process to increase the proportion of rays that is landed on deck in good condition.

Acknowledgements

The following contributors were indispensable for our research into discards survival of rays: Owners, skippers and crews of all the participating fishing vessels for welcoming researchers with all their equipment on board and assisting them whenever needed; Mulder Transport BV for the transport of survival units from the fishing vessels to our laboratory in Yerseke; Visserijinnovatie Centrum Zuidwest Nederland for transporting all equipment to the fishing vessels and for maintaining all equipment in proper condition. PO Urk and Nederlandse Vissersbond for acquisition of the participating vessels.

References

1. Heithaus MR, Frid A, Wirsing AJ, Worm B. Predicting ecological consequences of marine top predator declines. Trends Ecol Evol. 2008 Apr 1;23(4):202-10.
2. Stevens JD, Bonfil R, Dulvy NK, Walker PA. The effects of fishing on sharks, rays, and chimaeras (chondrichthyans), and the implications for marine ecosystems. ICES J Mar Sci. 2000 Jun 1;57(3):476-94.
3. Schindler DE, Essington TE, Kitchell JF, Boggs C, Hilborn R. Sharks and tunas: fisheries impacts on predators with contrasting life histories. Ecol Appl. 2002 Jun;12(3):735-48.
4. Ellis JR, Clarke MW, Cortés E, Heessen HJL, Apostolaki P, Carlson JK, Kulka DW, Payne A, Cotter J, Potter T. Advances in Fisheries Science: 50 Years on from Beverton and Holt. 2008.
5. Heessen H, Ellis J. Haaien en roggen in de Noordzee. De Levende Natuur. 2009 Jan 1;110(6):257-60.
6. Walker PA, Heessen HJ. Long-term changes in ray populations in the North Sea. ICES J Mar Sci. 1996 Dec 1;53(6):1085-93.
7. Sguotti C, Lynam CP, García-Carreras B, Ellis JR, Engelhard GH. Distribution of skates and sharks in the North Sea: 112 years of change. Glob Chang Biol. 2016 Aug;22(8):2729-43.
8. ICES. Working group on elasmobranch fishes (WGEF). ICES Scientific Reports. 2022;4(74):848.
9. Van Overzee HM, Kraan ML, Quirijns FJ. Beleidsondersteunend onderzoek haaien en roggen. IMARES; 2014.
10. Batsleer J, Bleeker K, Brunel T, van Hal R, Staat L. Overzicht beschikbare gegevens ten behoeve van Nederlandse beleidsdoelen voor haaien en roggen. Wageningen Marine Research rapport C114.20. 2020.

11. Batsleer J, Griffiths CA, Bleeker K, Johnston G, Cardinale M, Lorance P. Comparisons of landings to scientific advice indicates overshooting within the common TAC for skates and rays in the Northeast Atlantic. ICES J Mar Sci. 2024.
12. European Union. Regulation (EU) No 1380/201308 of the European Parliament and of the Council of 11 December 2013 on the Common Fisheries Policy. Official Journal of the European Union. 2013; 354: 22–61.
13. Schram E, Molenaar P. Discards survival probabilities of flatfish and rays in North Sea pulse-trawl fisheries. Wageningen Marine Research; 2018.
14. Schram E, van de Pol L, Molenaar P, van Mens A, Bleeker K, Gazi KM, van Dalen P, Suykerbuyk W, Batsleer J. Survival probabilities of thornback and spotted rays discarded by beam trawl and flyshoot fisheries. Wageningen Marine Research; 2023.
15. Kaiser MJ, Spencer BE. Survival of by-catch from a beam trawl. Mar Ecol Prog Ser. 1995 Oct 5;126:31-8.
16. Enever R, Catchpole TL, Ellis JR, Grant A. The survival of skates (Rajidae) caught by demersal trawlers fishing in UK waters. Fish Res. 2009 Apr 1;97(1-2):72-6.
17. Depestele J, Desender M, Benoît HP, Polet H, Vincx M. Short-term survival of discarded target fish and non-target invertebrate species in the "eurocutter" beam trawl fishery of the southern North Sea. Fish Res. 2014 Jun 1;154:82-92.
18. Breen M, Catchpole T. ICES guidelines for estimating discard survival. ICES cooperative research report/Rapport des Recherches Collectives de l'ICES. 2021 Sep 1;351.
19. Reintroduction, invasive species specialist groups' taskforce on moving plants, animals for conservation purposes. Guidelines for reintroductions and other conservation translocations. IUCN; 2013.
20. Van Bogaert N, Ampe B, Uhlmann SS, Torreele E. Discard survival estimates of commercially caught skates of the North Sea and English Channel. Report of the Project Sumaris. 2020 Sep 22.
21. Van der Reijden KJ, Molenaar P, Chen C, Uhlmann SS, Goudswaard PC, van Marlen B. Survival of undersized plaice (Pleuronectes platessa), sole (Solea solea), and dab (Limanda limanda) in North Sea pulse-trawl fisheries. ICES J Mar Sci. 2017 Jul 1;74(6):1672-80.
22. Schram E, Molenaar P, Goedhart PW, Poos JJ. Effects of environmental conditions and catch processing on survival probability of plaice discards in the North Sea pulse trawl fishery. Plos One. 2023 Jun 14;18(6):e0287020.
23. Kaplan EL, Meier P. Nonparametric estimation from incomplete observations. J Am Stat Assoc. 1958 Jun 1;53(282):457-81.

24. Harrington DP, Fleming TR. A class of rank test procedures for censored survival data. Biometrika. 1982 Dec 1;69(3):553-66.
25. Uhlmann SS, Theunynck R, Ampe B, Desender M, Soetaert M, Depestele J. Injury, reflex impairment, and survival of beam-trawled flatfish. ICES J Mar Sci. 2016 Mar 1;73(4):1244-54.
26. Raby GD, Packer JR, Danylchuk AJ, Cooke SJ. The understudied and underappreciated role of predation in the mortality of fish released from fishing gears. Fish Fish. 2014 Sep;15(3):489-505.
27. Gosch M, Hernandez-Milian G, Rogan E, Jessopp M, Cronin M. Grey seal diet analysis in Ireland highlights the importance of using multiple diagnostic features. Aquat Biol. 2014 Apr 2;20(2):155-67.
28. Gosch M, Cronin M, Rogan E, Hunt W, Luck C, Jessopp M. Spatial variation in a top marine predator's diet at two regionally distinct sites. PLoS One. 2019 Jan 2;14(1):e0209032.
29. Morizur Y, Berrow SD, Tregenza NJ, Couperus AS, Pouvreau S. Incidental catches of marine-mammals in pelagic trawl fisheries of the northeast Atlantic. Fish Res. 1999 Jul 1;41(3):297-307.
30. Hill BJ, Wassenberg TJ. The probable fate of discards from prawn trawlers fishing near coral reefs: A study in the northern Great Barrier Reef, Australia. Fish Res. 2000 Oct 1;48(3):277-86.
31. Davis MW. Key principles for understanding fish bycatch discard mortality. Can J Fish Aquat Sci. 2002 Nov 1;59(11):1834-43.
32. Cook KV, Reid AJ, Patterson DA, Robinson KA, Chapman JM, Hinch SG, Cooke SJ. A synthesis to understand responses to capture stressors among fish discarded from commercial fisheries and options for mitigating their severity. Fish Fish. 2019 Jan;20(1):25-43.
33. Parker SJ, Rankin PS, Hannah RW, Schreck CB. Discard mortality of trawl-caught lingcod in relation to tow duration and time on deck. N Am J Fish Manag. 2003 May 1;23(2):530-42.
34. Mandelman JW, Farrington MA. The physiological status and mortality associated with otter-trawl capture, transport, and captivity of an exploited elasmobranch, Squalus acanthias. ICES J Mar Sci. 2007 Jan 1;64(1):122-30.
35. Somerton DA, Otto RS, Syrjala SE. Can changes in tow duration on bottom trawl surveys lead to changes in CPUE and mean size?. Fish Res. 2002 Mar 1;55(1-3):63-70.
36. Veldhuizen LJ, Berentsen PB, De Boer IJ, Van De Vis JW, Bokkers EA. Fish welfare in capture fisheries: A review of injuries and mortality. Fish Res. 2018 Aug 1;204:41-8.
37. Braccini M, Van Rijn J, Frick L. High post-capture survival for sharks, rays and chimaeras discarded in the main shark fishery of Australia? 2012 Feb. PloS one 27;7(2):e32547.

38. Uhlmann SS, Ampe B, Van Bogaert N, Berghe CV, Vanelslander B. Flatfish survivors have tales to tell: Cold seawater and reduced deployment duration contribute to the survival of European plaice (Pleuronectes platessa) discarded by Belgian beam trawlers. Fish Res. 2021 Aug 1;240:105966.

39. Mandelman JW, Cicia AM, Ingram Jr GW, Driggers III WB, Coutre KM, Sulikowski JA. Short-term post-release mortality of skates (family Rajidae) discarded in a western North Atlantic commercial otter trawl fishery. Fish Res. 2013 Mar 1;139:76-84.

Figure 1ab

Click here to access/download;Figure;Fig 1ab SurvCurve_Beam trawl_2023-11-16.tif

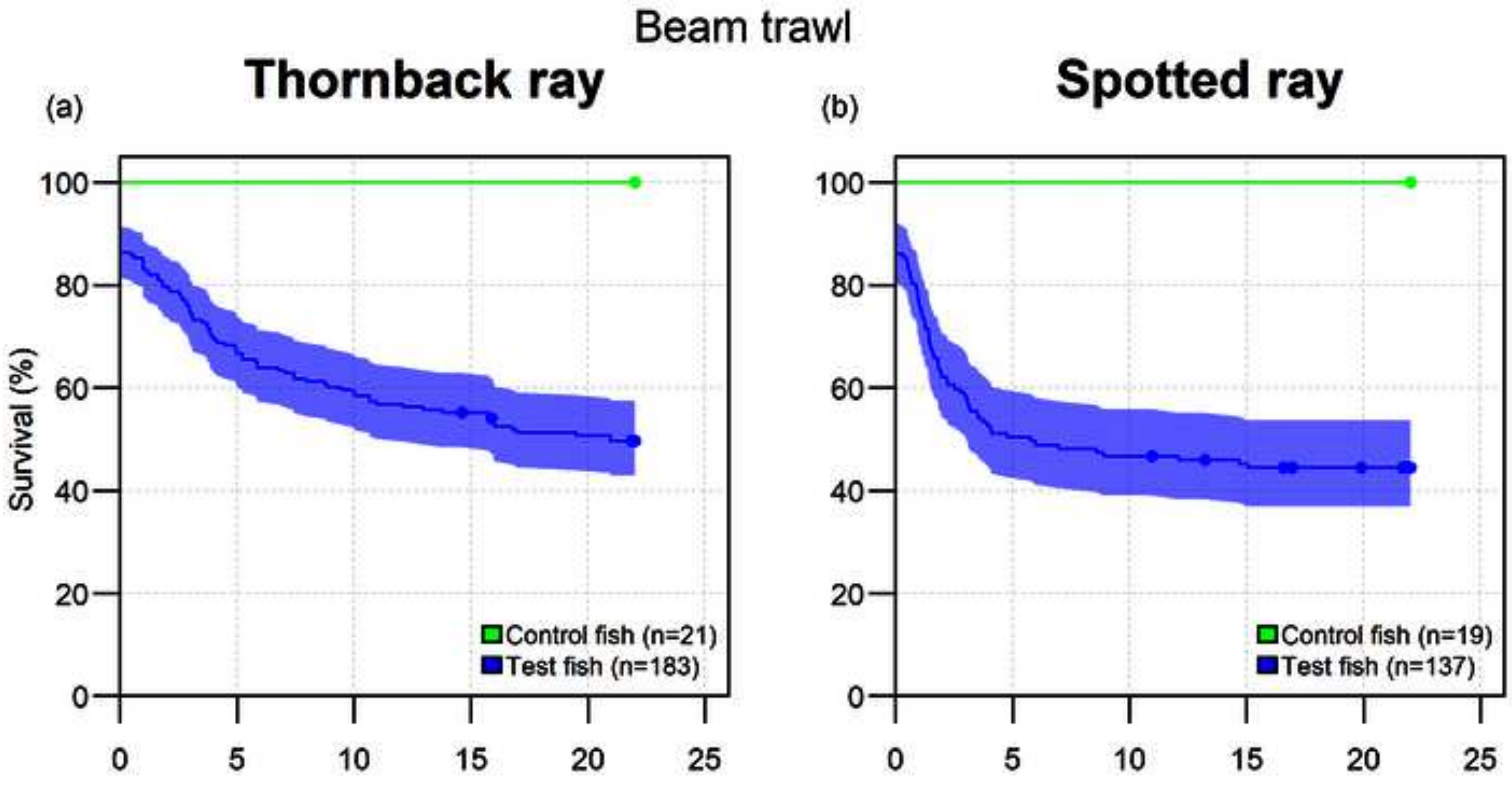

Figure 1c

Click here to access/download;Figure;Fig1c SurvCurve_Pulse trawl_2023-11-16.tif

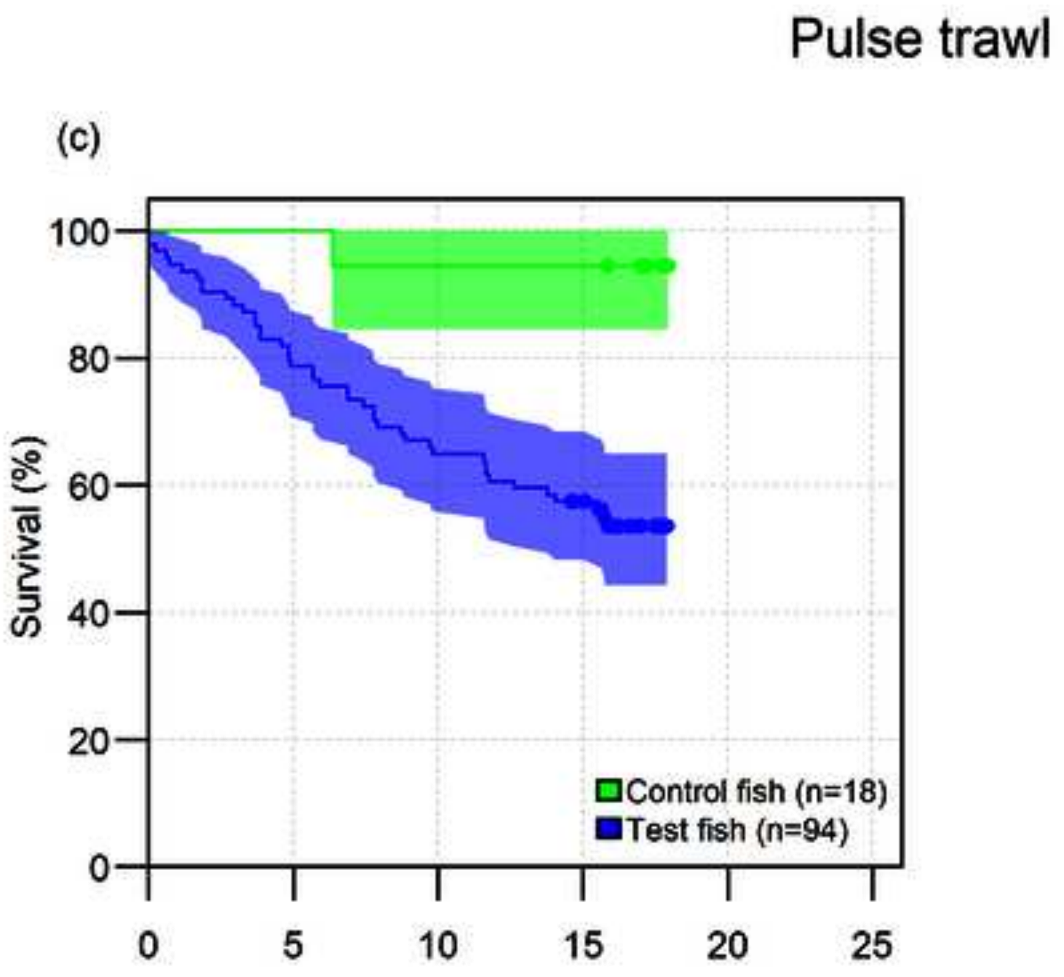

Click here to access/download;Figure;Fig 1de SurvCurve_Flyshoot_2023-11-16.tif

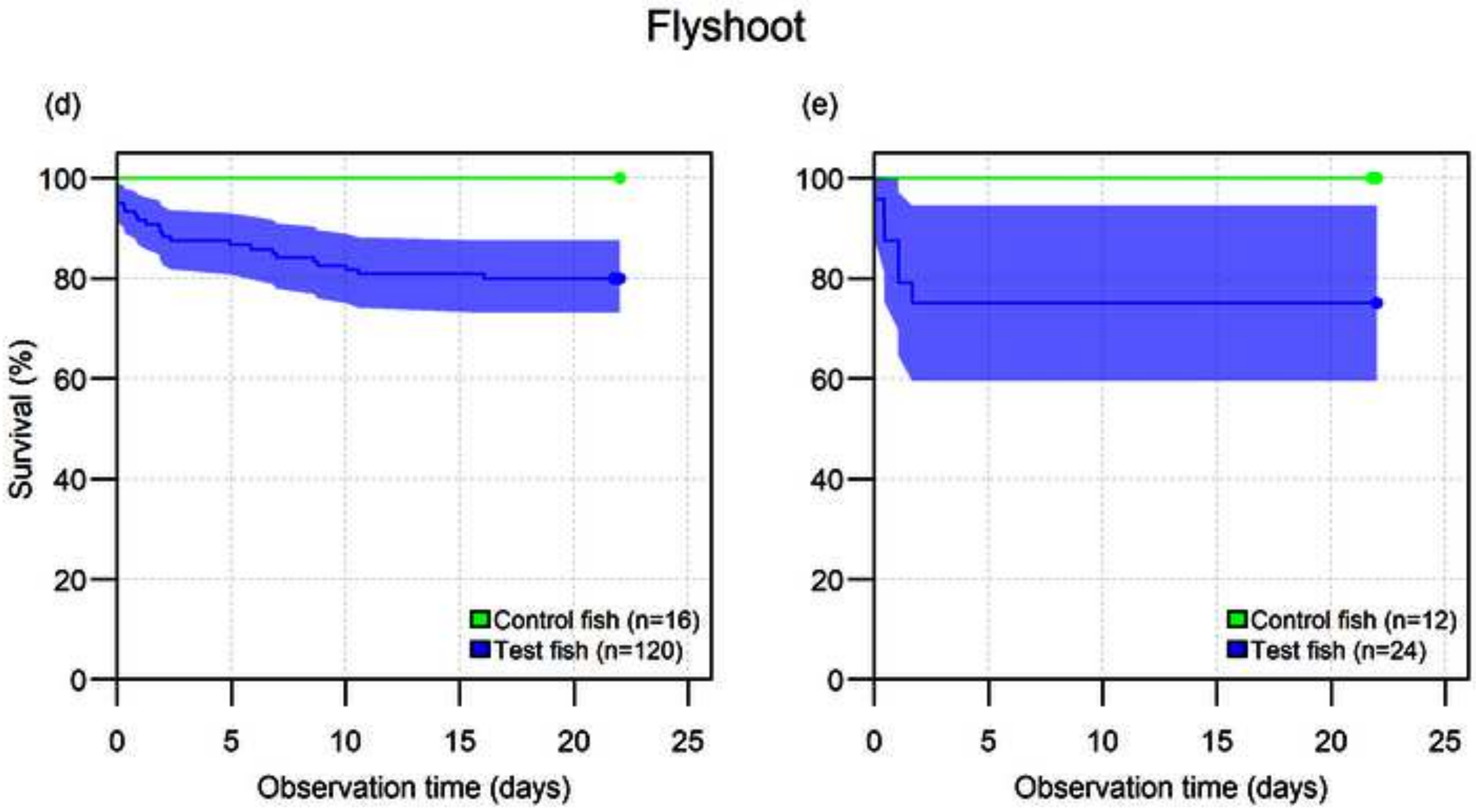

Click here to access/download;Figure;Fig 2a Survival by gear.tif

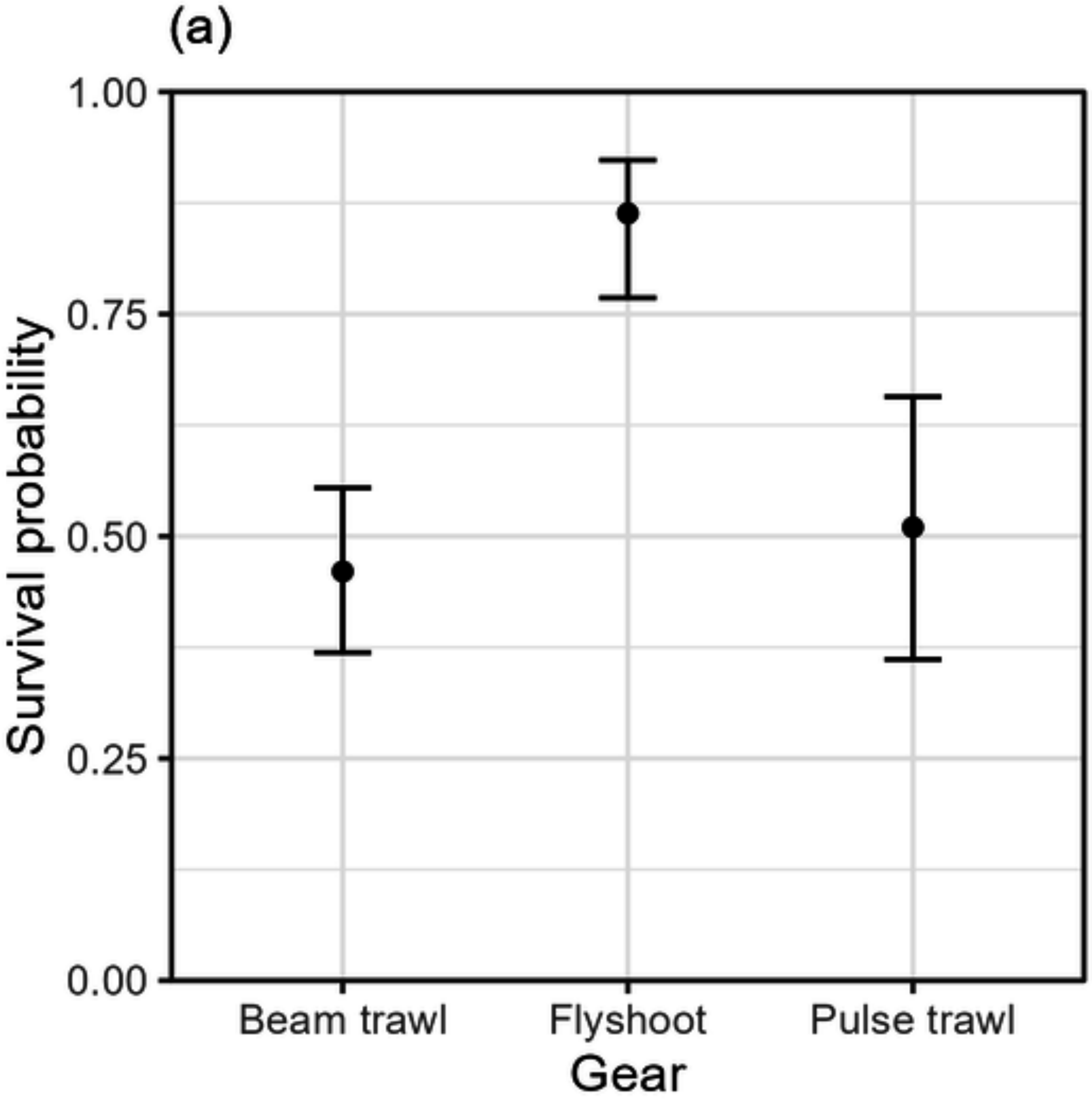

Fig 2b

Click here to access/download;Figure;Fig 2b Survival by temperature.tif

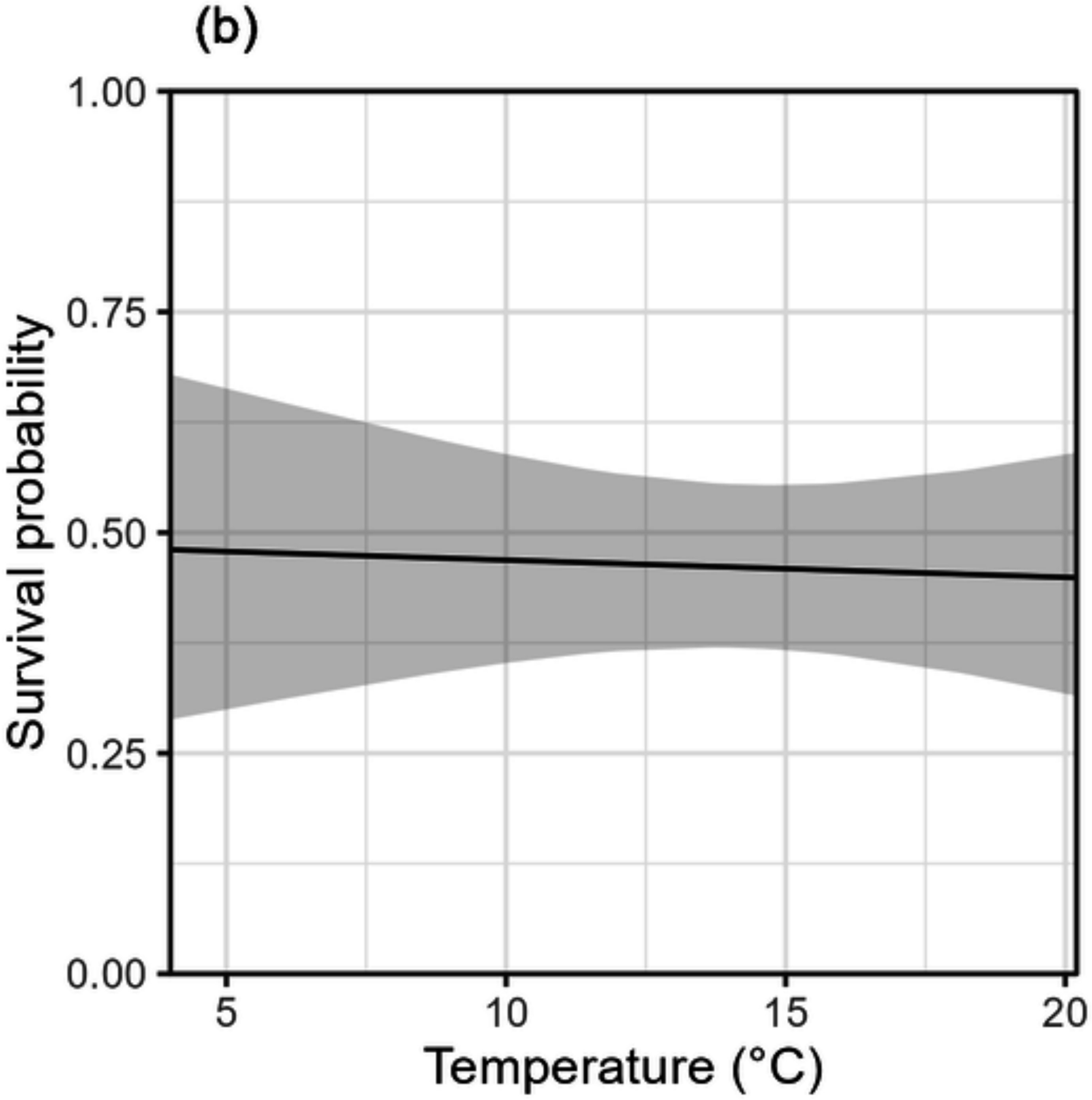

Fig 2c

Click here to access/download;Figure;Fig 2c Survival by catch processing time.tif

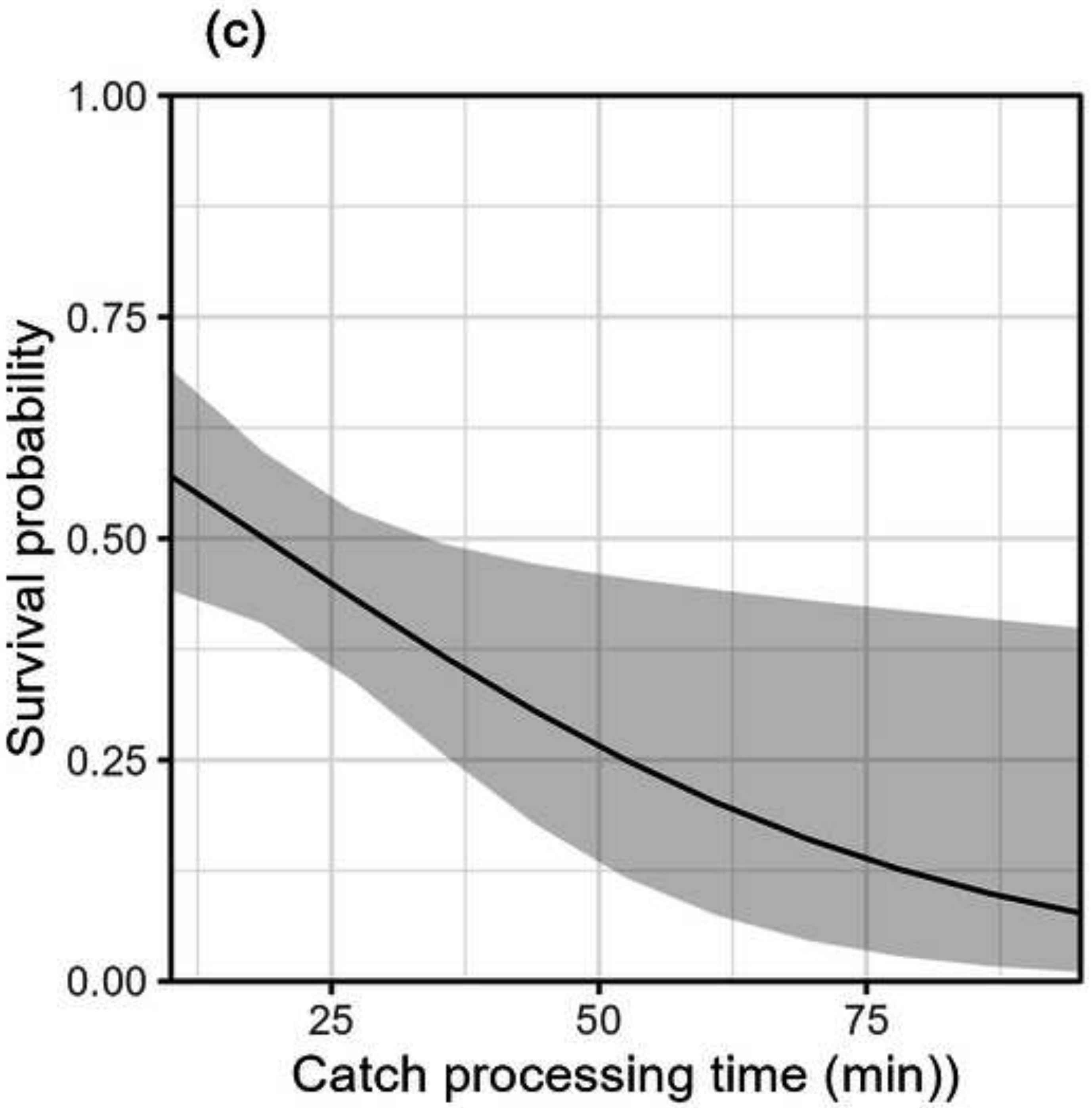

Click here to access/download;Figure;Fig 2d Survival by wave height.tif

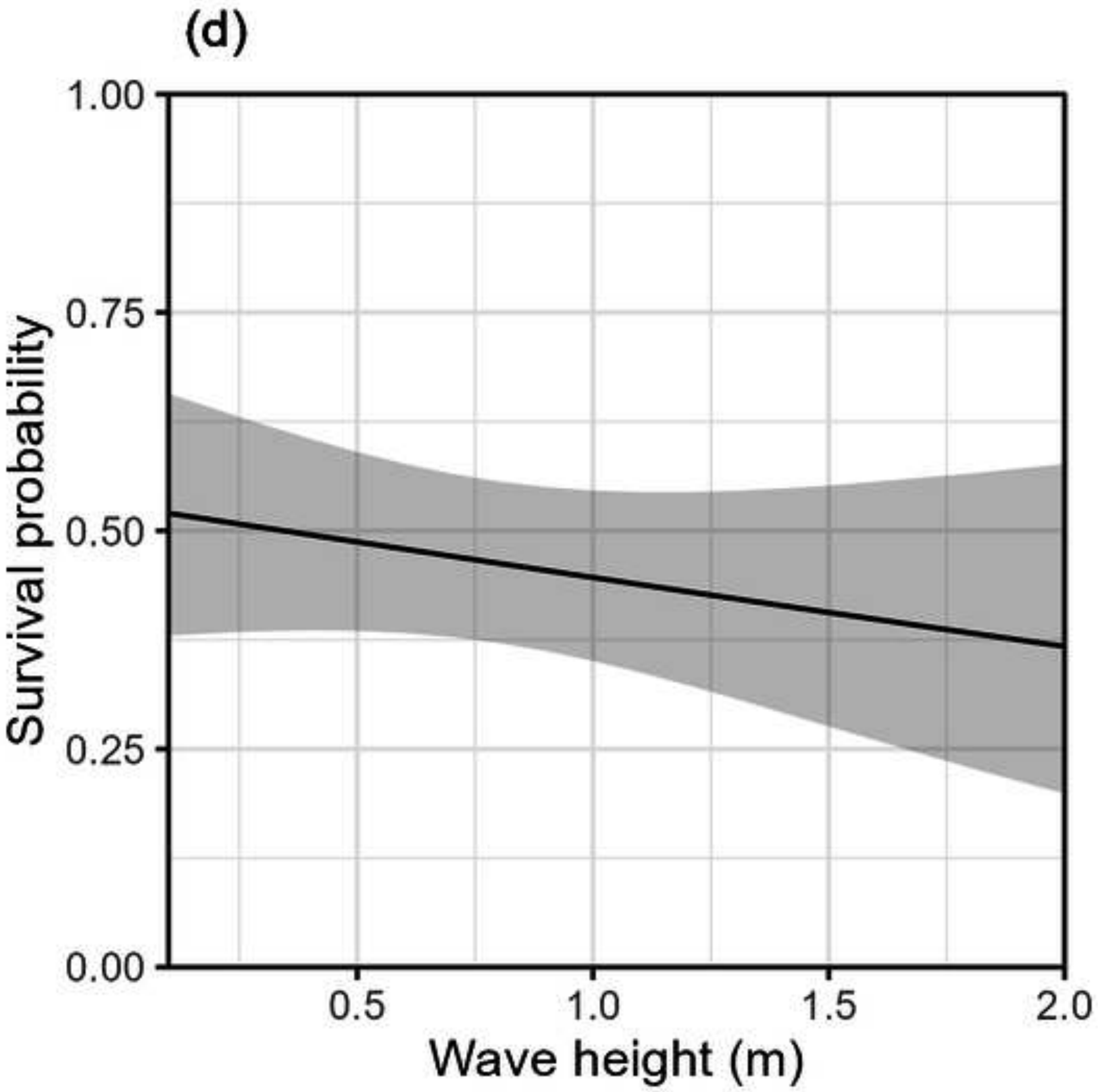

Fig 2e

Click here to access/download;Figure;Fig 2e Survival by temperature and wave height.tif

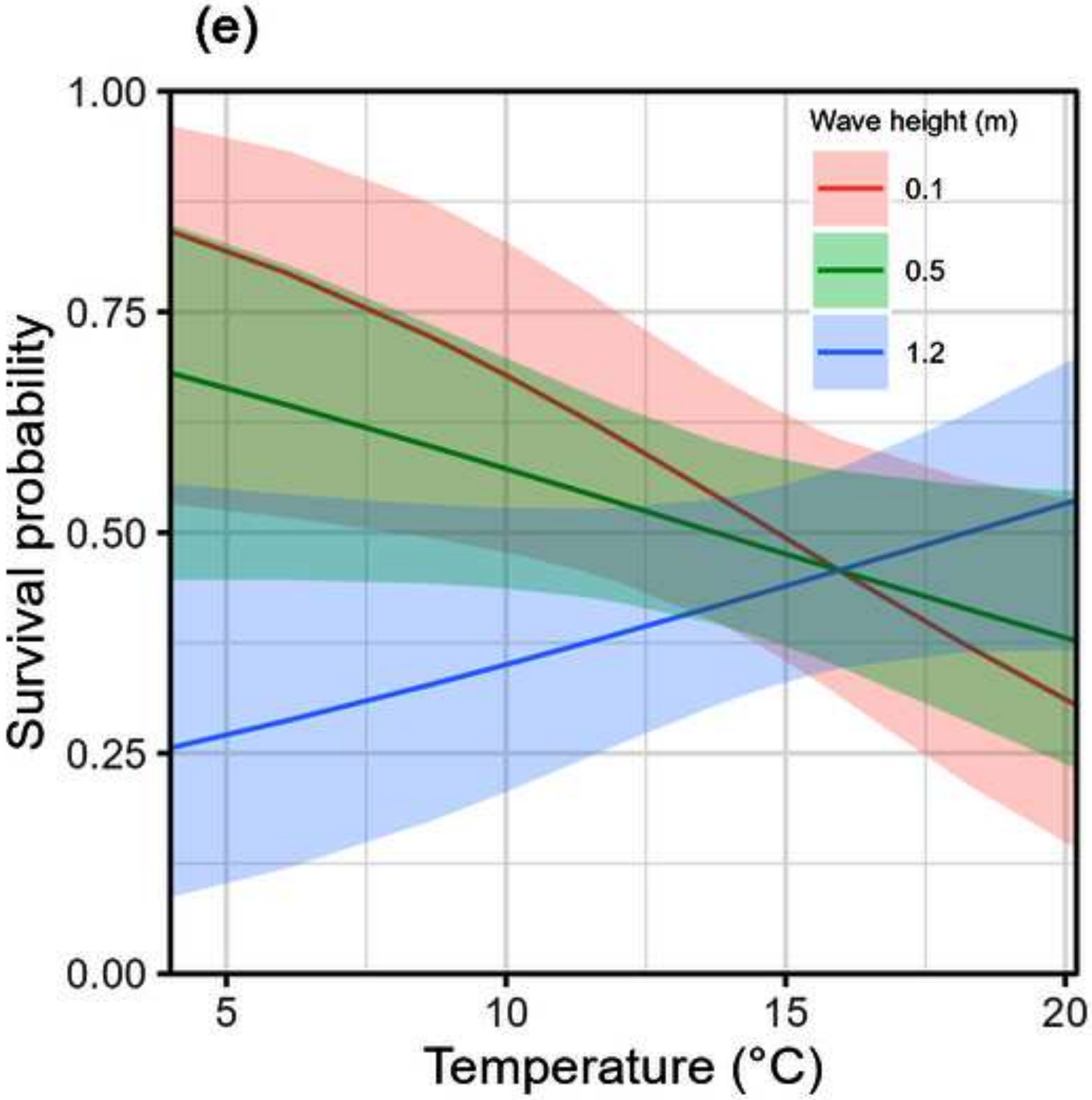

Click here to access/download;Figure;Fig 3a
SurvCurveCond_Beam_pulse trawl2023-12-21.tif

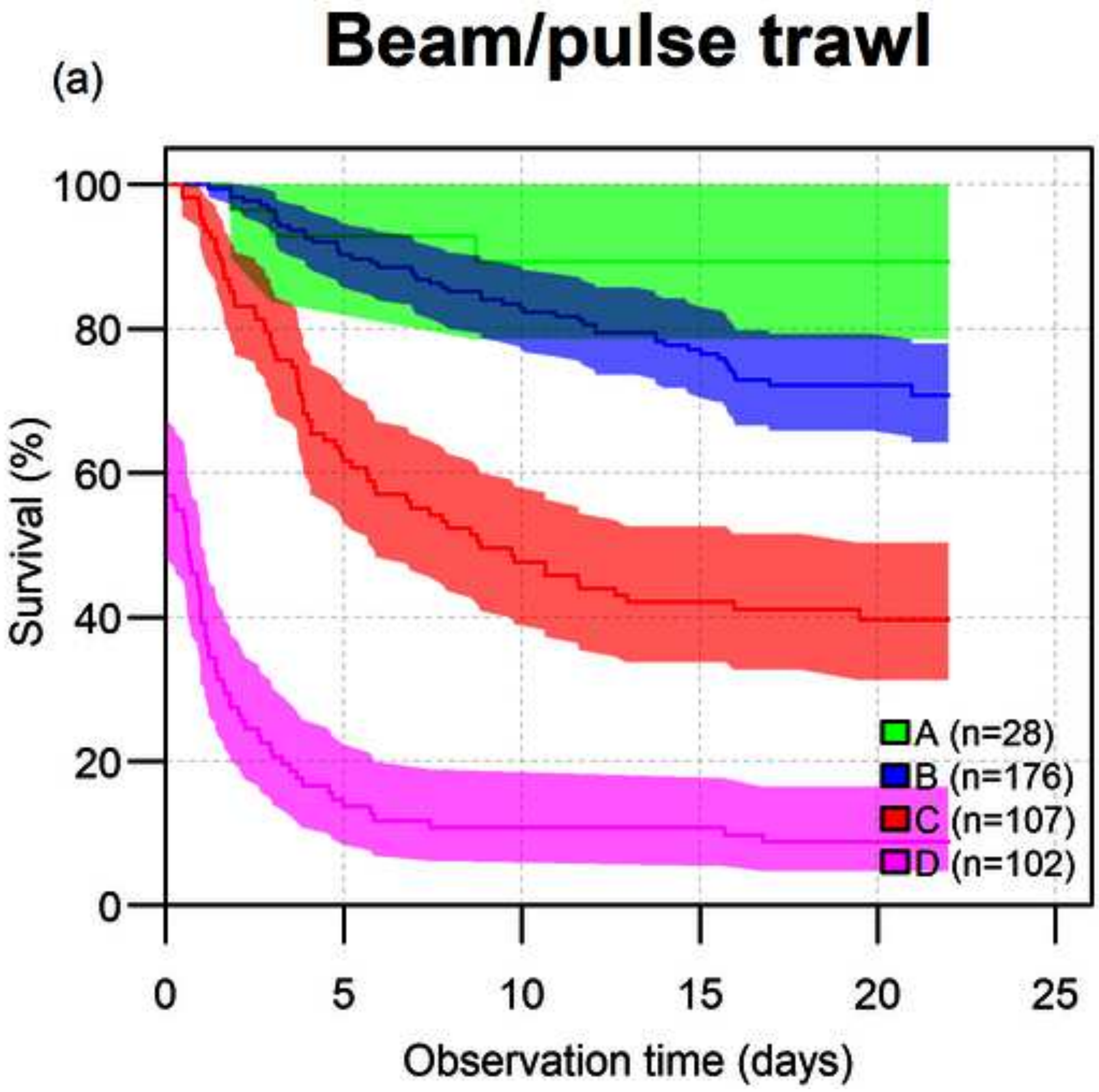

Fig 3b
Click here to access/download;Figure;Fig 3b SurvCurveCond_Flyshoot2023-12-21.tif

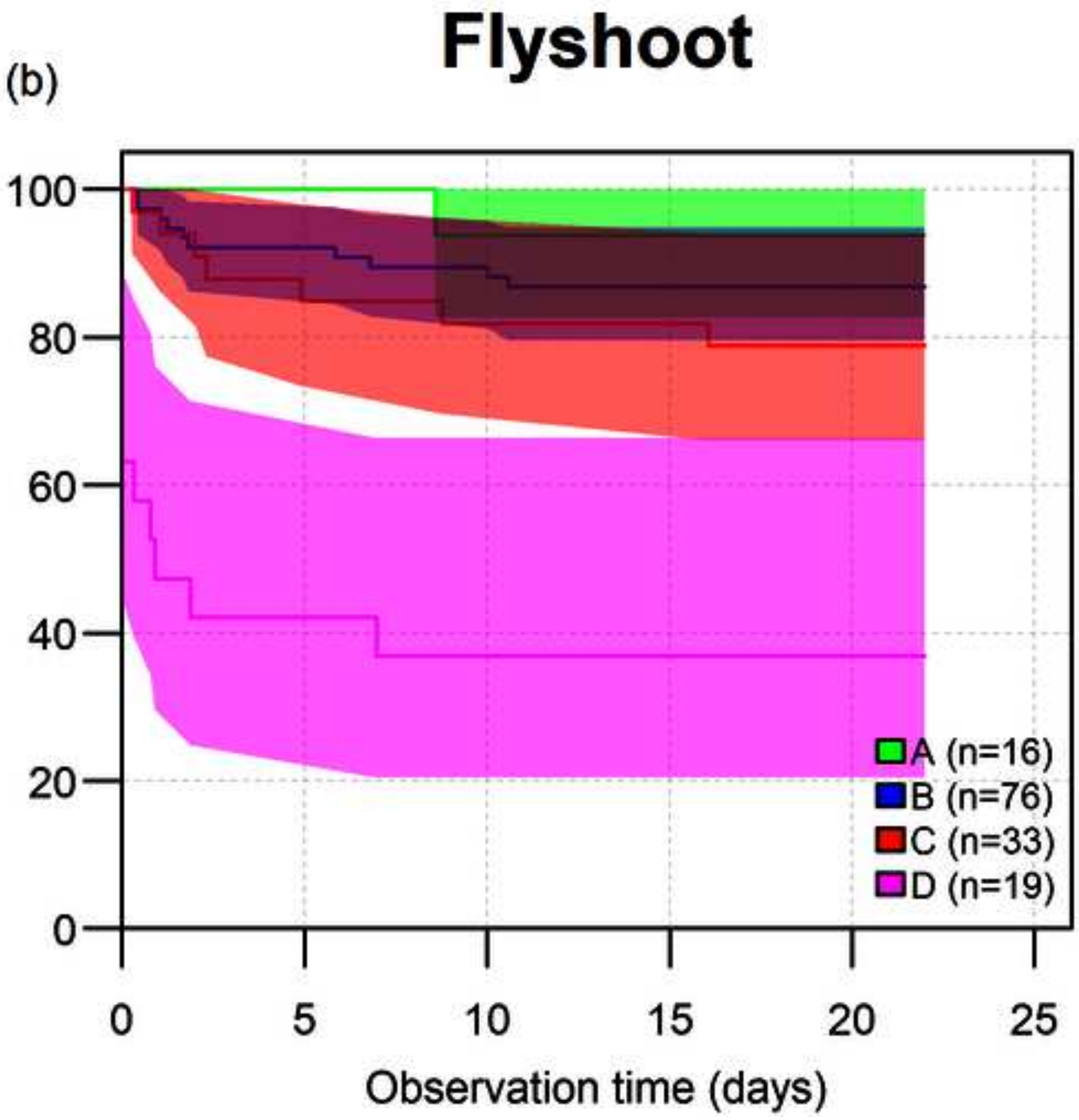

Click here to access/download
Supporting Information
SI ray discards survival PLoSOne.docx
