# Supplementary material for: Survival probabilities of thornback skate (Raja clavata) and spotted skate (Raja montagui) discarded by tickler chain beam trawl, pulse trawl, and flyshoot fisheries
Source: PLoS One. 2024 Dec 19;19(12):e0314032. doi: 10.1371/journal.pone.0314032 (PMC11658617; doi:10.1371/journal.pone.0314032)
Supplement: Attachment — Submitted filename: PONE-D-24-20479 Response to reviewer comments.docx [file pone.0314032.s007.docx]

**Rebuttal**

**PONE-D-24-20479**

**Survival probabilities of thornback and spotted rays discarded by tickler chain beam trawl, pulse beam trawl and flyshoot fisheries**

Note: reference to line numbers concerns the line numbers in the original manuscript. As revision led to changes in line numbers, referred line numbers may not correspond with line numbers as they appear in the revised documents.

**Journal Requirements**

|  | **Requirement** | **Author response** | **Changes to MS** |
| --- | --- | --- | --- |
| 1 | Please ensure that your manuscript meets PLOS ONE's style requirements, including those for file naming. The PLOS ONE style templates can be found at <https://journals.plos.org/plosone/s/file?id=wjVg/PLOSOne_formatting_sample_main_body.pdf> and  <https://journals.plos.org/plosone/s/file?id=ba62/PLOSOne_formatting_sample_title_authors_affiliations.pdf> | Done according to instructions | Various changes. |
| 2 | To comply with PLOS ONE submissions requirements, in your Methods section, please provide additional information regarding the experiments involving animals and ensure you have included details on (1) methods of sacrifice, (2) methods of anesthesia and/or analgesia, and (3) efforts to alleviate suffering. | Methods of sacrifice, of anaesthesia and/or analgesia were not used. No efforts to alleviate suffering were employed.  Therefore no details on these aspects were included in the Methods section. | Re. (1) & (3) we added this sentence to the Ethics statement:  “Humane end points nor alleviations of suffering were applied as these would interfere with survival time measurements.” |
| 3 | We note that the grant information you provided in the ‘Funding Information’ and ‘Financial Disclosure’ sections do not match. When you resubmit, please ensure that you provide the correct grant numbers for the awards you received for your study in the ‘Funding Information’ section. |  | We updated the funding information. |
| 4 | Thank you for stating the following financial disclosure:    "European Union, European Maritime and Fisheries Fund (EMFF)"  Please state what role the funders took in the study.  If the funders had no role, please state: "The funders had no role in study design, data collection and analysis, decision to publish, or preparation of the manuscript. If this statement is not correct you must amend it as needed.  Please include this amended Role of Funder statement in your cover letter; we will change the online submission form on your behalf. |  | We included the statement in the cover letter cf. your instructions. |
| 5 | Please provide a complete Data Availability Statement in the submission form, ensuring you include all necessary access information or a reason for why you are unable to make your data freely accessible. If your research concerns only data provided within your submission, please write "All data are in the manuscript and/or supporting information files" as your Data Availability Statement. | We included the data as S4 and wrote "All data are in the manuscript and/or supporting information files" as our Data Availability Statement. | Table S4 with data included. |
| 6 | Please include captions for your Supporting Information files at the end of your manuscript, and update any in-text citations to match accordingly. Please see our Supporting Information guidelines for more information: <http://journals.plos.org/plosone/s/supporting-information>. |  | Included according to instructions |
|  |  |  |  |

**Reviewer #1**

|  | **Comment** | **Author response** | **Changes to MS** |
| --- | --- | --- | --- |
| 1 | Introduction and Background:  Your introduction needs to provide a broader context for the study. The concept of discard survival should be framed within the larger scope of fisheries management and conservation. | We agree with the reviewer now mention the larger scope for fisheries management and conservation. | The following text was added to the introduction (line 61-62):  Part of the discarded skates may not survive and information their fate is important for modelling skate population dynamics for conservation and stock assessment purposes … |
|  | Additionally, it is crucial to cite foundational works that enhance the understanding of discard survival and the factors influencing it. In particular, the studies by Tiralongo et al. (2018) and Tiralongo et al. (2020) are fundamental for comprehending biological discards and survival, emphasizing that survival can sometimes be species-specific, as seen with Dasyatis pastinaca. Integrating these references will significantly strengthen your background section. See the attached pdf for further details. | We agree with the reviewer that enhancing the understanding of discard survival by citing foundational works benefits our manuscript. We chose to include Clarke et al. (2024) and Elliot et al. (2020). Clarke et al. (2024) provides discard survival estimates for the same species as used in our study, in the Atlantic. The main differences between their study and ours are the kind of fishing gear studied, which leads to important insights in interactive effects of gear and species on discards survival. Elliot et al. (2020) discusses the relevance of discard survival in the light of sustainable management of skates. | The factors influencing discards survival are now touched upon in the Introduction, mainly to stress the need for carefully designed experiments to obtain results that are representative for the species, gears and conditions of main interest. |
| 2 | Discussion:  The discussion needs to integrate your findings with existing literature more robustly. Again, referencing Tiralongo et al. (2018, 2020) will provide a deeper understanding of how species-specific traits influence discard survival. | We carefully considered the articles suggested by the reviewer. The suggested papers do not provide information on how species-specific traits influence discard survival, so we cannot use them for that purpose. Instead we refer to Clarke et al (2024) as their research on fixed nets provides important insights in interactive effects of gear and species on discards survival. | We expanded the section on the effects of species and size. |
|  | Discuss the implications of gear type on survival probabilities in greater detail and compare your findings with other studies on different species or in different regions. | We agree with the reviewer that the effect of gear should be discussed in more detail. Note that the implications of gear type are already discussed in line 378-392. The only gear effect detected was the higher survival in flyshoot fisheries. As mentioned this effect can most likely be explained by the shorter time fish spend in the net and the cleaner catches. We compare our results to other relevant studies for fisheries with comparable gears.  We expanded the discussion on the effect of gear type by including a study on fixed nets (Clarke et al., 2024). The main insight is that effects of variables on survival are not necessarily generic but may interact with gear type. We mention this only briefly as this was already pointed out by Clarke et al. (2024). | We expanded the section on the effects of species and size and briefly discuss the apparent interaction with gear type. |
|  | Provide a more comprehensive analysis of the interaction between water temperature, wave height, and survival probabilities. Explore why these factors might influence survival and how they interact. This analysis will add depth to your discussion and provide valuable insights. | The interaction between temperature and wave height is discussed in line 424-438, in our opinion as far as our data and relevant literature allows. We see no leads to elaborate this discussion without becoming too speculative. We would like to emphasize that in our field study a balanced experimental design re. the levels and numbers of observations for environmental and operational variables conditions is not possible. We consequently can only explore the effects of variables on survival and should be prudent in drawing conclusions. | None |
| 3 | Conclusion:  Your conclusion should be strengthened by explicitly stating the practical applications of your findings for fisheries management. How can these results be used to improve sustainability practices? | We agree with the reviewer that such statement should be added to the manuscript. We now state that these results can improve sustainability practices as they support an exemption to the landing obligation (prevent unnecessary fishery mortality) and support more sustainable exploitation as stock assessment may benefit from more accurate fishery mortality estimates.  However, we choose to add this text to the Discussion rather than the Conclusion as we prefer to limit the conclusions to the data collected in this study. | The following text was added at the end of the Discussion:  “The results of this study can contribute to more sustainable exploitation and conservation of skates in European waters. The relatively high survival probabilities may make skates eligible for exemptions to the landing obligation, effectively preventing 100% mortality among skates destined to be landed instead of discarded under the landing obligation. The availability of representative discards survival estimates may benefit skate stock assessments as it provides better insight in the contribution of discarding to fishery mortality.” |
|  | Additionally, suggest areas for future research, particularly those that could address any limitations identified in your study or expand on the interaction effects observed. | We agree with the reviewer. In fact we already recommended research on gear modifications that reduce stressors, but this was not clearly phrased as recommendation. We changed the text accordingly.  We also add the recommendation to collect more data on survival under variable conditions. | We changed the phrasing in the conclusion to make clear that focus on gear modification is a recommendation for future research.  We also added this sentence at the end of the conclusion:  “Additional data collection on survival probabilities under variable environmental and operational conditions will increase our understanding of the interactive effects of these conditions and support further decrease of mortality among skate discards.” |
| **Line** | ***Line specific comments*** | | |
| 46 | The authors must explain the difference between skate and ray and decide which term to use. In particular Raja clavata and R. montagui are skates.  Please check all the text | We agree with the reviewer. The two species in our study are skates and not rays. Therefore we decided to use skate instead of ray throughout the MS. This way all text is taxonomically correct. In our opinion there is no need to explain the difference between skates and rays because rays were not included in our study. | “ray” changed into “skate” throughout the MS.  Order and class added after first mentioning skates (line 37)  “Skates and rays” changed into “skates” as rays were not part of the study. |
| 49 | Not in Italic | Agree | Changed as suggested |
| 388-391 | Both introduction and discussion are only focalized on the study area. You must further discuss on a "wide range" on discards for batoids and survival for some species. Please, consider this two recent and relevant work:  Tiralongo et al., 2018: Discards of elasmobranchs in a trammel net fishery targeting cuttlefish, Sepia officinalis Linnaeus, 1758, along the coast of Sicily (central Mediterranean Sea). Regional Studies in Marine Science.  Tiralongo et al., 2021: Biological Aspects of Juveniles of the Common Stingray, Dasyatis pastinaca (Linnaeus, 1758) (Elasmobranchii, Dasyatidae), from the Central Mediterranean Sea. Journal of Marine Science and Engineering. | We agree with the reviewer that the scope of Introduction and Discussion should be somewhat broader and made changes accordingly.  However, we still mainly focus on the same or similar species in comparable fisheries to maintain a clear focus.  Please note that we carefully considered the suggested papers and concluded that in our opinion they do not provide additional understanding of discards survival relevant to our manuscript because they do not study survival probabilities of discards nor factors influencing this. | We expanded the Introduction and Discussion as mentioned above. |
| 398 | This can be also related to the type of species: see the manuscript of Tiralongo et al., 2018 and 2020 mentioned above. | We agree with the reviewer that discards survival probability can vary among species. However in the current study we did not detect a significant species effect on survival. | We expanded the discussion on species effects as mentioned earlier. |
| 537 | In Italic | Agree | Changed as suggested |
| 569 | In Italic | Agree | Changed as suggested |
| 579 | In Italic | Agree | Changed as suggested |

**Reviewer #2**

| **Line** | **Comment** | **Author response** | **Changes to MS** |
| --- | --- | --- | --- |
| 1-2 | Please, add the scientific names of the species in the title | Agree | Changed as suggested |
| 18-21 | Too long sentence, please, modify. | Agree | Modified and now slightly shorter. |
| 28 | respectively | Disagree. We try to avoid “, respectively” for clear writing. | None |
| 49 | sp. is not in italics in the scientific world. | Agree | Changed as suggested |
| 52 | of total length? Disc width? Please, specify. | Agree | We changed  “restricted to skates larger than 45-55 cm”  into  “restricted to skates with total lengths above 45 to 55 cm” |

**Reviewer #3**

|  | **Comment** | | **Author response** | | **Changes to MS** |
| --- | --- | --- | --- | --- | --- |
| 1 | The only think I’m not clear on is how discard mortality was recorded (see line 115 comment). Was each skate that came through the catch categorized as dead or alive, or just the ones sampled for the survival study? If the latter is the case, the dead rays were included in the tank study, but not actually placed in a tank? | Mortality was recorded only for sampled skates. We have no information on skates that came through the catch but were not sampled. Indeed, skates that were considered dead when sampled (no spiracle movement for more than 15 seconds) were recorded as dead at time zero and not placed in tanks (line 160) | | See response to comment from Reviewer #3 on Line 115 below. | |
| **Line** | ***Line specific comments*** | | | | |
| 52 | What measurement for minimum size? Total length? Disc width? | | Total length | | To make this clear we changed  “restricted to skates larger than 45-55 cm”  into  “restricted to skates with total lengths above 45 to 55 cm” |
| 60 | Has any of these ray species been a choke species in a year? It’s good if they haven’t yet, but it would be good context to know how close these species have been to closing the fishery. | | No because since the implementation of the landing obligation there have been exemptions for skates. | | None |
| 69 | What was the survival rate of Van Bogaert et al? | | 56.9% | | We added the survival rate by changing  “Van Bogaert et al. [20] monitored survival up to 21 days post catch for a limited number of thornback rays (n=21).”  Into  “Van Bogaert et al. [20] monitored survival of a limited number of thornback skates (n=21) up to 21 days post catch and reports a survival probability of 56.9%.” |
| 115 | Was data recorded (alive or dead) for all rays that came onboard? I think it’s also worth a statement in this section that condition of the ray was not a factor in choosing it as a test-fish, except if the ray was already dead (no spiracle movement for 15 seconds). | | No we recorded only data for the subset of skates that were randomly sampled from catches.  Indeed, condition was not a factor in choosing a skate as test-fish; sampling was random. Some of the randomly sampled skates were dead. See also response to the first comment of Reviewer #3.  We agree with the reviewer that this needs to be clarified better in this section. | | To make the text more clear we made the following changes:  The sentence in Line 160 “Fish that were considered dead … and not placed in tanks”.  Was replaced by:  “Sampled skates that showed no spiracle movement for more than 15 seconds during condition assessment were recorded as dead at time zero and not placed in tanks.”  We added this sentence at the end of the section on test-fish collection:  “All skate sampling was random and irrespective of their condition. Samples were subsets of the total amount of skates appearing on the sorting belts and we only recorded data for these subsets.” |
| 127 | Why did you focus on the big ones? Isn’t the minimum size 45-55 cm? Why 65 cm? | | Because quota limitations often force fishers to discard skates above the minimum sizes. Therefore we wanted to include skates above minimum sizes in our samples. | | We inserted the following sentence in Line 107:  “Although landings are currently restricted to skates with total lengths above 45 to 55 cm, larger skates were not excluded from the samples because quota limitations may force fishers to discard larger specimens.” |
| 130 | I recommend moving this sentence to Line 126. | | Agree | | Moved as recommended by the reviewer. |
| 266 | Wasn’t the maximum time in a tank 21 days? I’m hesitant to put too much value in a leveling out of mortality at 20 days of a 21 day experiment. Looking at Fig 1a and 1c, I wouldn’t say the mortality rate has leveled off. This doesn’t change your results, just what you say about them. | | For skates monitored in the lab for 21 days (beam trawl and flyshoot) the minimum time in a tank was 22 days for skates sampled on day 4 at sea (Thursday) and transferred to the lab on one day later (Friday). The maximum time in a tank was 25 days for skates sampled on day 1 (Monday) at sea (line 170).  We agree with the reviewer that in Fig.1a and c mortality may not have levelled off. Fig 1c was our first experiment(Pulse trawl) and we mention in the Discussion that based on these first results we decided to extent the monitoring in the lab for 14 to 21 days.  The survival curves Fig1 ab and 1de were all truncated at the minimum monitoring period of 22 days. This does not affect our results because none of the skates that was monitored for 23 up to 25 days died after day 22 but it does affect the extend to which curves level off. We therefore updated the curves and they now cover the full length of the monitoring periods. This ‘improves’ the levelling off in Fig. 1a, no mortality after day 21. We cannot exclude that a longer monitoring period would not have affected the results but probably any effect would have been small. | | Figs1 ab and 1de were updated and now cover the full monitoring period of 25 days instead of being truncated at day 22.  We added this sentence to the Discussion:  “This resulted in survival monitoring for 22 to 25 days post catch for individual skates depending on the day of sampling at sea. Despite the extended monitoring period, we cannot exclude that mortality of thornback skate sampled from beam trawl fisheries did not level out completely. We therefore consider our discards survival probability estimate for thornback skate caught by beam trawl fisheries a maximum value and recommend a minimum monitoring period of 25 days post catch for thornback skate.” |
| 268 | I’m not sure how direct mortality was recorded. Was every ray examined for direct mortality before discard? Or was this only of sampled rays? If the latter was the case, did you sample another ray from that haul to get to target numbers? See Line 115 comment also. | | Direct mortality (based on > 15s no operculum movement) was recorded only for sampled skates.  Skates that were considered dead were not placed in tanks (line 160) but recorded as dead at T=0 and part of the targeted numbers. We did however seize the opportunity of tanks not being used because of skates being dead at T=0 to sample more skates than originally targeted (Line 128). | | See response to comment on Line 115. |

**Changes made by authors**

| **Line** | **Change** | **Justification** |
| --- | --- | --- |
| 7-8 | Added Jan Jaap Poos as co-auteur. | Because of his essential contributions to data analysis and interpretation. |
| 64 | Changed “beam trawl fisheries” into “beam trawl fisheries targeting sole” | To further specify the type of fisheries and ensure that it is clear that we do not refer to other types of beam trawls such as shrimp trawls. |
| 64 | Added “also known as Scottish seine” after “flyshoot” | The common name for this type of fisheries is flyshoot among Dutch fishers. However, internationally this fisheries is also referred to as Scottish seine. This is now added for the sake of clarity. |
| 416 | Added: “and do not sustain injuries due to collisions caused by sloshing during rough seas” | This potential negative effect should be mentioned here |
