# Supplementary material for: Survival probabilities of thornback skate (Raja clavata) and spotted skate (Raja montagui) discarded by tickler chain beam trawl, pulse trawl, and flyshoot fisheries
Source: PLoS One. 2024 Dec 19;19(12):e0314032. doi: 10.1371/journal.pone.0314032 (PMC11658617; doi:10.1371/journal.pone.0314032)
Supplement: Attachment — Submitted filename: PONE-D-24-479_R2 Response to reviewer comments.docx [file pone.0314032.s010.docx]

**Response to reviewer comments PONE-D-24-20479_R1**

**Reviewer #1**

|  | **Comment** | **Author response** | **Changes to MS** |
| --- | --- | --- | --- |
| 1 | Extend this section, too short. Require to be more general. Quote the manuscript Tiralongo et al., 2018.  Discards of elasmobranchs in a trammel net fishery targeting cuttlefish, Sepia officinalis Linnaeus, 1758, along the coast of Sicily (central Mediterranean Sea) | The reviewer refers the Line 37-44, the first paragraph of the Introduction.  We agree with the reviewer and expanded this paragraph with more general information on skates in relation to fisheries. | First paragraph of the Introduction was expanded. See track-changes. |
| 2 | If you are not sure about the species, you can't use the common name of "common skate", use "skate" only please, followed by the genus sp. | It only became clear around 2010 that the species referred to as common skate is actually two distinct species: flapper skate (*Dipturus intermedius*) and blue skate (*Dipturus flossada*). Because the two species cannot be visually distinguished they are still referred to as common skate complex by ICES or common skate (*Dipturus* sp.)  We agree with the reviewer that is be best to mention both species instead of *Dipturus* sp. and would suggest to change the text accordingly. However, following a comment by Reviewer #3 the text mentioning common skate has been deleted. | The text mentioning common skate has been deleted. |
| 3 | sp. is not in italic | Agree | The text mentioning common skate has been deleted. |
| 4 | Conclusions are not an extreme synthesis of Discussion, you should use a more generalist approach to the issue, e.g. survival of elasmobranchs in fisheries. | We agree with the reviewer that the broader context of our results needs to be addressed. We therefore added the last paragraph to the Discussion following the first round of review. We there state that our results can improve sustainability practices as they support an exemption to the landing obligation (prevent unnecessary fishery mortality) and support more sustainable exploitation as stock assessment may benefit from more accurate fishery mortality estimates.  However, we disagree with the reviewer that this should be included in the Conclusion as the conclusions should be limited to the data collected in this study. | None. |

**Reviewer #2**

|  | **Comment** | **Author response** | **Changes to MS** |
| --- | --- | --- | --- |
| 1 | The authors must explain the difference between skate and ray and decide which term to use. In particular Raja clavata and R. montagui are skates.  Please check all the text. | We agree with the reviewer. The two species in our study are skates and not rays. Therefore we decided to use skate instead of ray throughout the MS. This way all text is taxonomically correct. In our opinion there is no need to explain the difference between skates and rays because rays were not included in our study. | “ray” changed into “skate” throughout the MS. |
| 2 | *Sp* Not in Italic | agreed | Changed as suggested |
| 3 | Pleuronectes platessa, Limanda limanda in Italic | agreed | Changed as suggested |
| 4 | Squalus acanthias in Italic | agreed | Changed as suggested |

**Reviewer #3**

|  | **Comment** | **Author response** | **Changes to MS** |
| --- | --- | --- | --- |
| 1 | Line 49-51: You state that starry and common skates are discarded. Is this still true after the landing obligation came into effect in 2019. Do these two species have exemptions for the landing obligation? This is not clear in this paragraph. | Starry and common skates are prohibited species and therefore exempted from the landing obligation.  We conclude that mentioning these species here is redundant and only causes confusion. We therefore decided to delete the text on starry and common skate. | Text on starry and common skate was deleted. |
| 2 | I recommend adding the species that currently have exemptions from the landing obligation at the end of the paragraph (Line 57) or the next paragraph (Line 64). | We agree and added a sentence after Line 57 as suggested by the reviewer.  Please note that the EU regulation does not specify the species, so neither can we.  Note that the update of the reference numbering following the inserting of 2 extra references is not visible as track-changes. | The following sentence was added after Line 57:  Currently all fisheries in Western waters [13] and the North Sea [14] are exempted from the obligation to land their entire catches of skates that fall under the common TAC [11]. The exemptions do not specify the species and expire in 2027. |
| 3 | Line 78: In the abstract you include that survival probabilities are “affected by gear, catch processing time, wave height, and the interaction between water temperature and wave height.” I think it’s worth including the that you investigated predictors of survival as an objective. | We agree with the reviewer and included this objective. | The yellow marked text was added.  The objectives of this study were to measure survival probabilities of skates discarded by tickler chain and pulse beam trawl fisheries and flyshoot fisheries and to explore which variables predict survival probability. |
| 4 | Line 120: remove the third ‘belt’ in this line since I think you mean “manually collected by crew members.” | Thanks for noting our mistake. ‘belt’ was unintentionally included here and should be deleted. | ‘belt’ was deleted |
| 5 | Line 128: Why do you use a cutoff of 65 cm rather than 55 cm. I understand why you want large individuals, but why the 10 cm difference? | The cut off at 65 cm was determined by the maximum size at which we learned that skates could be housed in Type 1 monitoring units (see experimental facilities). This cut off determined the onboard housing capacity for skates > 65 cm and has nothing to do with the MLS of 55 cm.  We acknowledge that this causes confusion and therefore added an extra clarifying sentence. | The sentence below was added in line 131.  Note that the cut-off at > 65 cm TL was dictated by the tank size of the type 1 on-board monitoring units (see below) and is unrelated to the MLS of skates. |
| 5 | Line 138-140: Please rewrite this sentence to enhance clarity. I think the point of this sentence is that you were able to sample more skates when skates died while at sea and sometimes you were also able to put two skates in one tank. | The reviewer is correct re. the point of this sentence. We agree that this could be phrased more clear. | The original sentence:  Skates that were dead when sampled or died at sea, and housing of two small skates (< 25 cm) in one tank were used as opportunities to sample more skates per trip.  Was replaced by:  We sampled more skates per trip than aimed for in case skates were dead when sampled or died at sea and when two small skates (< 25 cm) could be housed in one tank. |
